# Development of Integrated Vectors with Strong Constitutive Promoters for High-Yield Antibiotic Production in Mangrove-Derived *Streptomyces*

**DOI:** 10.3390/md22020094

**Published:** 2024-02-18

**Authors:** Mingxia Zhao, Zhiqiang Yang, Xinyue Li, Yaqi Liu, Yingying Zhang, Mengqian Zhang, Yangli Li, Xincheng Wang, Zixin Deng, Kui Hong, Dongqing Zhu

**Affiliations:** Key Laboratory of Combinatorial Biosynthesis and Drug Discovery, Ministry of Education, School of Pharmaceutical Sciences, Wuhan University, Wuhan 430071, China; zmingxia2021@163.com (M.Z.); nilealexed@gmail.com (Z.Y.); lixinyue0880@163.com (X.L.); liuyaqi2019@163.com (Y.L.); yingying199603@163.com (Y.Z.); zhangmengqian0331@163.com (M.Z.); 13512775882@163.com (Y.L.); ww709693222@163.com (X.W.); zxdeng@sjtu.edu.cn (Z.D.)

**Keywords:** marine *Streptomyces*, integrative vector, constitutive promoter, elaiophylin, azalomycin F, armeniaspirol

## Abstract

It is important to improve the production of bioactive secondary products for drug development. The *Escherichia coli—Streptomyces* shuttle vector pSET152 and its derived vector pIB139 containing a strong constitutive promoter *ermE*p* are commonly used as integrative vectors in actinomycetes. Four new integrative vectors carrying the strong constitutive promoter *kasO*p*, *hrdB*p, *SCO5768*p, and SP44, respectively, were constructed and proven to be functional in different mangrove-derived *Streptomyces* host strains by using kanamycin resistance gene *neo* as a reporter. Some biosynthetic genes of elaiophylins, azalomycin Fs, and armeniaspirols were selected and inserted into these vectors to overexpress in their producers including *Streptomyces* sp. 219807, *Streptomyces* sp. 211726, and *S*. *armeniacus* DSM 43125, resulting in an approximately 1.1–1.4-fold enhancement of the antibiotic yields.

## 1. Introduction

Natural products are critical sources of drug resources. *Streptomyces* strains, harboring complex secondary metabolic gene clusters, are the most important producers of antibiotics and other bioactive secondary metabolites. The *Escherichia coli*—*Streptomyces* shuttle vector pSET152 [1] and its derived vector pIB139 [2,3] containing strong constitutive promoter *ermE*p* are commonly employed in high-yield strain breeding. They are non-replicative in streptomycetes but integrate into the chromosome to yield stable recombinant strains, thus avoiding the possible problems associated with autonomously replicating plasmids. pSET152 and pIB139 were widely used in gene function analysis, secondary metabolite biosynthetic gene cluster mining, and silent gene cluster activation [4,5,6]. They also introduced some homologous or heterologous genes into *Streptomyces* to increase antibiotic production [7,8,9,10].

*ermE*p* is a mutated promoter of the erythromycin resistance gene of *Saccharopolyspora erythrea* [2]. During our research, we found that the *ermE*p* did not express a high level in some *Streptomyces* spp. It is necessary to enrich molecular tools for gene manipulation by constructing new pSET152-derived vectors with other strong constitutive promoters. *kasO*p* and *hrdB*p, an engineered promoter of the SARP family regulator gene and a native promoter of the principal sigma factor gene in *S. coelicolor*, constitutively transcribe gene expression more strongly than the *ermE*p* [11,12]. *SCO5768*p is also a strong constitutive promoter scanned from the *Streptomyces* species, which was twice as strong as *ermE*p* in *S. venezuelae* [13]. SP44 is a synthesized constitutive promoter, which was twice as strong as *kasO*p* in *S. avermitilis* [14]. As we all know, the promoter strength comparisons were performed using particular genes in special strains under specific conditions, so these findings should not be extrapolated to draw general conclusions.

In this work, we constructed four pSET152-derived vectors with the strong constitutive promoters reported [15], such as *kasO*p*, *hrdB*p, *SCO5768*p, and SP44. Kanamycin resistance gene *neo* was used as a reporter to test their transcriptional levels in *Streptomyces* strains. These vectors were applied in gene overexpression to increase the antibiotic production of the mangrove-derived *Streptomyces* strains, including elaiophylin producer *Streptomyces* sp. 219807 [16], azalomycin F producer *Streptomyces* sp. 211726 [17,18,19], and armeniaspirol producter *S. armeniacus* DSM 43125 [4,20].

## 2. Results

### 2.1. Construction of Recombinant Plasmids Harboring Strong Constitutive Promoters and the Corresponding Reporter Plasmids

**Figure 1:**
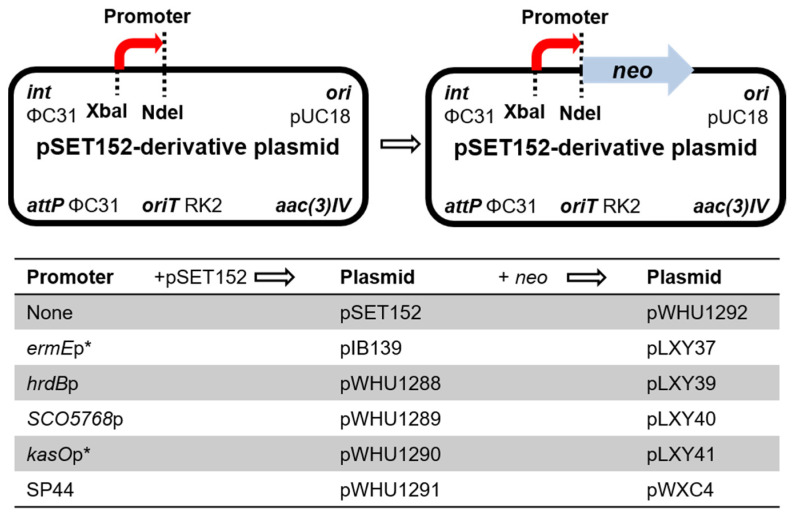
Genetic map of the base vectors.

The native promoters *hrdB*p, *SCO5768*p, and *kasO*p* were amplified by PCR, and the synthetic promoter SP44 was synthetized to develop four new integrating vectors pWHU1288-pWHU1291 (Figure 1). The construction of the following plasmids is listed in Table S1. Agarose gel electrophoresis analysis of PCR products or recombinant plasmids digested with restriction enzymes is shown in Figures S1 and S2.

Next, we need a reporter gene to test whether the four promoters express normally in *Streptomyces* strains. The kanamycin resistance gene *neo* was amplified from plasmid pHZ1358 [21] and inserted into pIB139 to generate plasmid pLXY37; the *neo* gene is controlled by the promoter *ermE*p*. pLXY37 was conjugated into *Streptomyces* strains, including *S. coelicolor* M145, *S. lividans* TK24, *S. albus* J1074, and *S. venezuelae* ISP5230. The recombinant strains and their corresponding wild-type strains were inoculated in TSBY liquid medium containing 0–50 μg/mL kanamycin and cultured at 30 °C for 48 h. The recombinant strains harboring pLXY37 showed obvious kanamycin resistance, as compared to the wild-type strains (Figure S3). The results proved the *neo* gene is functional, and can be used to reveal the strength of promoters.

The gene *neo* was also inserted into pSET152 to generate a promoter-less reporter plasmid pWHU1292 as the negative control. pWHU1292 was conjugated into *S. ceolicolor* M145 and the recombinant strain *S. ceolicolor* M145::pWHU1292 was inoculated in TSBY liquid medium containing 0–25 μg/mL kanamycin and cultured at 30 °C for 48 h. Compared with *S. ceolicolor* M145::pLXY37 as the positive control and the wild-type strain *S. ceolicolor* M145 as the negative control, *S. ceolicolor* M145::pWHU1292 was inhibited completely at the concentration of 18–25 μg/mL kanamycin (Figure S4). The results revealed that the *neo* gene can be used to test the promoter when the concentration of kanamycin is above 20 μg/mL. Then the *neo* gene was inserted into pWHU1288-pWHU1291, respectively, to generate the corresponding reporter plasmids pLXY39-pLXY41 and pWXC4 to test the strength of promoters in host strains (Figure 1).

### 2.2. Comparison of Promoter Strength in Different Streptomyces Strains

The reporter plasmids were conjugated into six different *Streptomyces* strains, including *S. coelicolor* M145, *S. lividans* TK24, *S. olivaceus* CGMCC 4.1369, *Streptomyces* sp. 219807, *Streptomyces* sp. 211726, and *S. armeniacus* DSM 43125, respectively. The kanamycin resistance level of the recombinant strains (Table 1 and Figures S5–S10) is a direct reflection of promoter activity.

In *S. coelicolor* M145, the strength of promoters is placed in the order of SP44 > *hrdB*p > *kasO*p*, *ermE*p* > *SCO5768*p. *S. lividans* and *S. coelicolor* are closely related species belonging to the *S. violaceouruber* sub-clade, but the promoter strengths in *S. lividans* TK24 and *S. coelicolor* M145 are different. The strength of promoters in *S. lividans* TK24 is placed in the order of SP44 > *SCO5768*p > *kasO*p* > *hrdB*p, *ermE*p*. Firstly, *SCO5768*p, the weakest promoter in *S. ceolicolor* M145, exhibited higher activity than the other natural promoters and ranked second only to the artificial promoter SP44 in *S. lividans* TK24. Secondly, contrary to the result in *S. ceolicolor* M145, the activity of *kasO*p* was higher than *ermE*p* and the expression level of *hrdB*p was similar to *ermE*p* in *S. lividans* TK24.

*S. olivaceus* is a member of glucose isomerase producers as food enzymes approved by the National Health Commission of the P. R. China (GB 2760-2014). The artificial promoter SP44 is also the most potent in *S. olivaceus* CGMCC 4.1369. The other promoters have similar expression levels.

In elaiophylin producer *Streptomyces* sp. 219807, the strength of promoters is placed in the order of SP44 > *hrdB*p, *kasO*p* > *ermE*p* > *SCO5768*p. The strength of promoters in azalomycin F producer *Streptomyces* sp. 211726 is placed in the order of SP44 > *hrdB*p > *kasO*p*, *ermE*p*, *SCO5768*p.

*S. armeniacus* DSM 43125 has been found to biosynthesize armeniaspirols, which are potent antibiotics against Gram-positive bacteria [4,22,23]. In *S. armeniacus* DSM 43125, *SCO5768*p and *kasO*p* show the best performances with kanamycin resistance up to 2200 μg/mL. The activities of SP44 (1800 μg/mL) are also superior to those of *ermE*p*. The strength of *hrdB*p is similar to *ermE*p*. The strength of promoters in *S. armeniacus* DSM 43125 is placed in the order of *kasO*p*, *SCO5768*p > SP44 > *hrdB*p, *ermE*p*.

In summary, the promoters SP44, *kasO*p*, and *hrdB*p showed better or similar activities compared with *ermE*p* in the *Streptomyces* strains tested. The strength of *SCO5768*p varies significantly among diverse strains. Next, we selected pWHU1288 containing *hrdB*p or pWHU1290 containing *kasO*p* to construct plasmids for overexpressing genes in *Streptomyces* sp. 219807, *Streptomyces* sp. 211726, and *S. armeniacus* DSM 43125.

### 2.3. Enhancement of Elaiophylin Production

**Figure 2:**
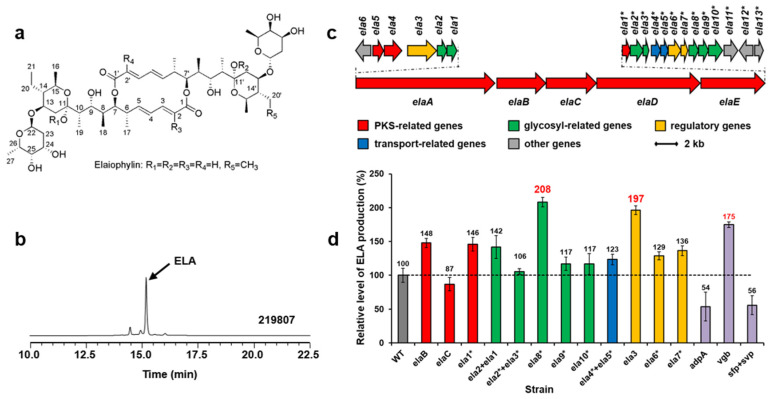
Structures of elaiophylins (**a**). HPLC analysis (252 nm) of extracts from *Streptomyces* sp. 219807 culture (**b**). ELA, elaiophylin. The elaiophylin biosynthetic gene cluster in *Streptomyces* sp. 219807 (**c**). Relative levels of elaiophylin production by *Streptomyces* sp. 219807 derivative strains detected and quantified by HPLC (**d**): WT, 219807::pWHU1288; *elaB*, 219807::pNN1; *elaC*, 219807::pDQ139; ela1*, 219807::pDQ137; *ela2* + *ela1*, 219807::pLXY45; ela2* + ela3*, 219807::pLXY44; ela8*, 219807::pLXY48; ela9*, 219807::pLXY50; ela10*, 219807::pLXY49; ela4* + ela5*, 219807::pLXY47; *ela3*, 219807::pLXY51; ela6*, 219807::pLXY52; ela7*, 219807::pLXY46; *adpA*, 219807::pLXY55; *vgb*, 219807::pLXY56; *sfp* + *svp*, 219807::pWHU2449. Error bars indicate the standard deviation (*n* = 3).

Elaiophylin (ELA) is a glycosylated macrodiolide antibiotic with various biological activities (Figure 2a). *Streptomyces* sp. 219807 is an elaiophylin producer from mangrove soil collected in Sanya of China [16] (Figure 2b). In order to obtain the biosynthetic gene cluster of elaiophylin, the whole genome of *Streptomyces* sp. 219807 was sequenced. A DNA region about 64-kb carrying 28 ORFs (Figure 2c, Table S3, accession no. PP236859) was believed to be involved in elaiophylin biosynthesis based on the proposed functions of the genes. Based on the reported elaiophylin biosynthetic gene cluster and biosynthetic pathway in other *Streptomyces* strains, the roles of biosynthetic genes in the cluster can be suggested (Figure S11).

Three polyketide synthases (PKS) genes (Figure 2c,d, red), seven sugar biosynthesis-related genes (green), two transfer-related genes (blue), and three regulator genes (yellow) in the cluster were amplified from the chromosome of *Streptomyces* sp. 219807 by PCR, inserted into pWHU1288 (the vector carrying *hrdB*p) to generate recombinant plasmids, which were conjugated in *Streptomyces* sp. 219807, respectively. The resulting recombinant strains were confirmed by PCR. The cultures of the derivative strains were extracted and tested by using HPLC and LC-MS. The results showed that the elaiophylin production of the *Streptomyces* sp. 219807 wild-type strain was about 0.98 g/L in this work and that the best two genes at increasing elaiophylin production were the NAD(P)-dependent oxidoreductase gene ela8* (about 2.04 g/L, increase of 108%) and the LuxR family transcriptional regulator gene *ela3* (about 1.93 g/L, increase of 97%). Then ela8* and *ela3* were selected and inserted into pWHU1291 (the vector carrying the promoter SP44) to generate recombinant plasmids, which were conjugated in *Streptomyces* sp. 219807 to increase the elaiophylin production further, respectively. However, the results showed that the production decreased about 30% unexpectedly (Figure S12a).

Three heterologous genes were also conjugated in strain *Streptomyces* sp. 219807, respectively, including AraC family transcriptional global regulator gene *adpA* from *S. coelicolor*, synthetic Vitreoscilla hemoglobin gene *vgb*, phosphopantetheinyl transferase (PPtase) gene *sfp* from *Bacillus subtilis*, and *svp* from *S. verticillus*. These genes have been proven to increase antibiotic production or activate silent gene clusters [7,8]. HPLC analysis showed that the overexpression of *vgb* increased the elaiophylin production about 75% (Figure 2d purple). The overexpression of *adpA* or *sfp* + *svp* decreased the elaiophylin production about 50%.

### 2.4. Enhancement of Azalomycin F production

**Figure 3:**
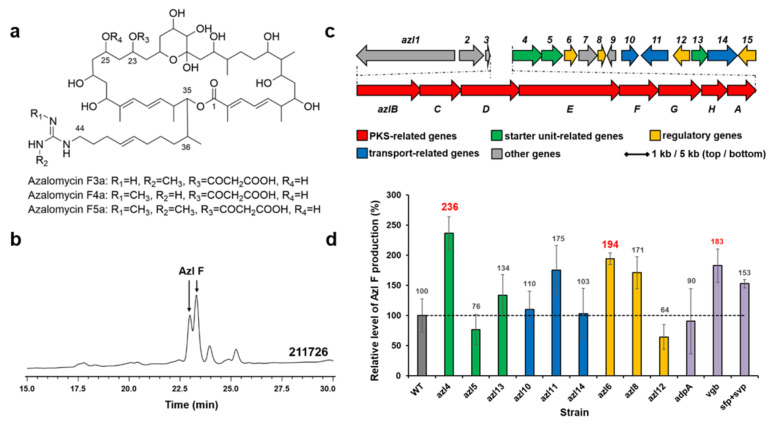
Structures of azalomycin Fs (**a**). HPLC analysis (241 nm) of extracts from *Streptomyces* sp. 211726 culture (**b**). Azl F, azalomycin F mixtures. The azalomycin biosynthetic gene cluster in *Streptomyces* sp. 211726 (**c**). Relative levels of azalomycin F production by *Streptomyces* sp. 211726 derivative strains detected and quantified by HPLC (**d**): WT, 211726::pWHU1288; *azl4*, 211726::pMX301; *azl5*, 211726::pMX302; *azl13*, 211726::pMX308; *azl10*, 211726::pMX305; *azl11*, 211726::pMX306; *azl14*, 211726::pMZ309; *azl6*, 211726::pMX303; *azl8*, 211726::pMX304; *azl12*, 211726::pMX307; *adpA*, 211726::pLXY55; *vgb*, 211726::pLXY56; *sfp* + *svp*, 211726::pWHU2449. Error bars indicate the standard deviation (*n* = 3).

Azalomycin F (Azl F) is a complex of ployhydroxy macrocyclic lactones with broad-spectrum antimicrobial activities (Figure 3a). *Streptomyces* sp. 211726 isolated from mangrove soil is a remarkable producer of azalomycin F (Figure 3b) [17,18]. A circa 130-kb DNA region carrying 23 ORFs (Figure 3c) was proved to be involved in azalomycin F biosynthesis [18,19]. Several genes, as shown in Figure 3c and d: four precursor biosynthesis-related genes (green), two transfer-related genes (blue), and three regulator genes (yellow) in the cluster, were amplified from the chromosome of *Streptomyces* sp. 211726 by PCR, and inserted into pWHU1288 to generate recombinant plasmids, which were conjugated in the strain *Streptomyces* sp. 211726 for overexpression, respectively. The derivative strains were fermented and the cultures were extracted and tested by using HPLC and LC-MS. Compared to the wild-type strain *Streptomyces* sp. 211726 as control (about 2.80 g/L), the best two genes increasing azalomycin F production were the 4-guanidinobutyryl-CoA ligase gene *azl4* (about 6.61 g/L, increase of 136%) and the TetR family transcriptional regulator gene *azl6* (about 5.43 g/L, increase of 94%), as shown in Figure 3d. pWHU1291 was also used to overexpress *azl4* and *azl6* in *Streptomyces* sp. 211726, respectively, in order to further improve the azalomycin F production. The analysis showed a decrease in production, similar to the results observed in *Streptomyces* sp. 219807 (Figure S12b).

The genes *adpA*, *vgb*, *sfp*, and *svp* were also conjugated in *Streptomyces* sp. 211726, respectively. HPLC analysis showed that the best gene increasing azalomycin F production was *vgb* (increase of 83%), as shown in Figure 3d (purple).

### 2.5. Enhancement of Armeniaspirol Production

**Figure 4:**
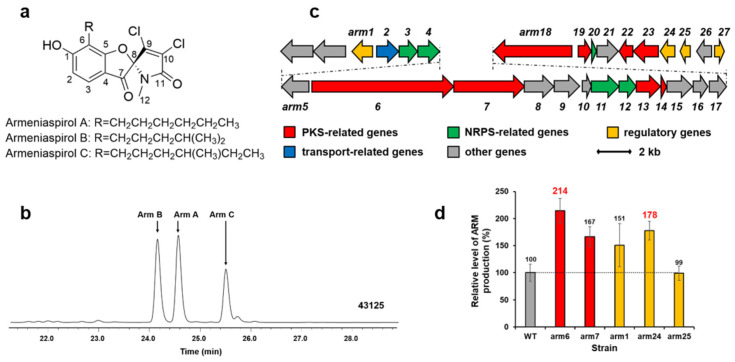
Structures of armeniaspirol (**a**). HPLC analysis (300 nm) of extracts from *S. armeniacus* DSM 43125 culture (**b**). Arm, armeniaspirol. The armeniaspirol biosynthetic gene cluster in *S. armeniacus* DSM 43125 (**c**). Relative levels of armeniaspirol production by *S. armeniacus* DSM 43125 derivative strains detected and quantified by HPLC (**d**): WT, 43125::pWHU1290; *arm6*, 43125::pZQ11; *arm7*, 43125::pZQ12; *arm1*, 43125::pYQ1; *arm24*, 43125::pYQ2; *arm25*, 43125::pYQ3. Error bars indicate the standard deviation (*n* = 3).

Armeniaspirols (Arm A, B, and C as shown in Figure 4a,b), with a unique chlorinated spiro[4.4]non-8-ene scaffold, are potent antibiotics against *Helicobacter pylori* and Gram-positive pathogens [20,22]. We cloned the armeniaspirol biosynthetic gene cluster from *S. armeniacus* DSM 43125 in previous work (Figure 4c) [4]. Two PKS genes (*arm6* and *arm7*) and three regulator genes (*arm1*, *arm24* and *arm25*) in the cluster were amplified from the chromosome of *S. armeniacus* DSM 43125 by PCR, and inserted into pWHU1290 (the vector carrying *kasO*p*) to generate recombinant plasmids, which were conjugated in the strain *S. armeniacus* DSM 43125 for overexpression, respectively. The cultures of *S. armeniacus* DSM 43125 and its derivative strains were extracted and tested by using HPLC and LC-MS. The results are shown in Figure 4d. The armeniaspirol production of the wild-type strain *S. armeniacus* DSM 43125 was 0.95 mg/L. The best two genes increasing armeniaspirol production were the PKS gene *arm6* (about 2.03 mg/L, increase of 114%) and the regulator gene *arm24* (about 1.69 mg/L, increase of 78%).

## 3. Discussion

Based on the comparison of promoter strength using kanamycin resistance gene *neo* as a reporter, SP44 is the strongest promoter in most of the tested strains with *S. armeniacus* being an exception. However, for unknown reasons, SP44 was ineffective in enhancing the yield of elaiophylin and azalomycin F. *kasO*p* and *hrdB*p showed similar or higher activity than the *ermE*p* in all the tested strains and they also had good performance in the improvement of antibiotic production. The activity of *SCO5768*p was not detected in some strains and the reason remains unexplained. In summary, pWHU1288 harboring *hrdB*p and pWHU1290 harboring *kasO*p* are favorable choices for the genetic manipulation of *Streptomyces* species.

pWHU1288 and pWHU1290 were used in gene overexpression to increase the antibiotic production of three *Streptomyces* strains, and we obtained high-yielding strains compared with the wild-type strains. Gene ela8*, *azl4*, and *arm6* overexpression doubled elaiophylin, azalomycin F, and armeniaspirol production, respectively. They are all biosynthetic genes of their corresponding gene clusters, which suggests that the products of these genes may be rate-limiting enzymes in the biosynthetic pathway. The gene cluster for a secondary metabolite harbors variable amounts of biosynthetic genes. Identifying the gene responsible for the rate-limiting step is challenging, especially when dealing with a gene cluster containing numerous biosynthetic genes. Trying each one individually becomes unrealistic. Therefore, pathway-specific activator genes of a one-component regulatory system are viable choices. Gene *ela3* is the only one-component regulatory system gene of the elaiophylin biosynthetic gene cluster, and overexpression of *ela3* increased elaiophylin production about 97%. The same goes for azalomycin F biosynthesis and armeniaspirol biosynthesis. Overexpression of the histidine kinase gene or the response regulator gene of a two-component regulatory system individually did not explicitly improve antibiotic production in the elaiophylin producer and armeniaspirol producer. We also tried co-overexpression of two genes of a two-component regulatory system, but no apparent effects were observed in this work (not shown).

Vitreoscilla hemoglobin is an oxygen-binding protein that promotes oxygen delivery under low oxygen conditions to increase the efficiency of cell metabolism. Normally, oxygen supply is insufficient during the shake flask fermentation, so overexpression of *vgb* increased antibiotic production in *Streptomyces* sp. 219807 and *Streptomyces* sp. 211726, as expected. Gene *adpA* did not improve the biosynthesis of azalomycin Fs in *Streptomyces* sp. 211726 and even repressed the biosynthesis of elaiophylins in *Streptomyces* sp. 219807. AdpA is a global transcriptional activator triggering morphological differentiation and secondary metabolism in *Streptomyces*. However, genes repressed by AdpA were also reported [24]. It is reasonable that the expressions of secondary biosynthetic gene clusters are either unaffected or repressed by AdpA. Phosphopantetheinyl transferase catalyzes the conversion of the carrier proteins of polyketide synthases and nonribosomal peptide synthases from the *apo* form to the active form (*holo* form). Overexpression of the corresponding genes into actinomycete strains achieved a significantly high activation ratio at which strains produced new metabolites [8,25,26]. At the same time, the metabolites produced in some wild-type strains were either eliminated or diminished in their PPtase-overexpressing strains [8]. Thus, it was not inevitable that azalomycin F production went up and elaiophylin production went down when PPtase genes were overexpressed in the producers. In summary, gene *vgb* is a promising candidate for enhancing antibiotic production.

## 4. Materials and Methods

### 4.1. General Materials and Experimental Procedures

The bacterial strains and plasmids used in this work are listed in Table S1. Primer sequences are listed in Table S2. Reagents and solvents purchased from Sigma-Aldrich were of the highest quality available and were used without further purification. Restriction enzymes, T4 DNA ligase, and DNA polymerase were purchased from New England Biolabs and used according to the manufacturer’s specifications. DNA primers were synthesized by TsingKe Co. Ltd. (Wuhan, China). Growth media and conditions used for *E. coli* and *Streptomyces* strains and standard methods for handling *E. coli* and *Streptomyces* in vivo and in vitro were as described previously, unless otherwise noted. All DNA manipulations were performed following standard procedures. DNA sequencing was carried out at TsingKe Co. Ltd. (Wuhan, China). Genome sequencing of *Streptomyces* sp. 219807 was performed by BGI Co. Ltd. (Wuhan, China) using the Illumina HiSeq 2000 System. ORFs of the secondary metabolite biosynthetic gene clusters were identified using antiSMASH (http://antismash.secondarymetabolites.org, accessed on 22 July 2018) [27], FramePlot (http://nocardia.nih.go.jp/fp4/, accessed on 22 August 2018) [28], and secondFind (http://biosyn.nih.go.jp/2ndfind/, accessed on 22 August 2018). The NRPS-PKS online tools (http://www.nii.ac.in/~pksdb/sbspks/master.html, accessed on 11 September 2018) and (http://nrps.igs.umaryland.edu/, accessed on 11 September 2018) were used to analyze PKSs [29].

The construction of plasmids is listed in Table S1. The plasmids were transferred into *E. coli* ET12567/pUZ8002, and the unmethylated plasmid was conjugated into *Streptomyces* strains, using apramycin to select the respective exconjugants. The exconjugants were confirmed by PCR.

### 4.2. Determination of Promoter Strength in Streptomyces Strains

Spores of the *Streptomyces* strains were harvested and resuspended in sterile water, and optical density at 450 nm was measured and normalized to the same level. Kanamycin-resistant analyses were carried out using either method 1: the spore suspension was inoculated in the liquid TSBY medium (0.5% yeast extract, 3% tryptone soya broth, 10.3% sucrose, pH 7.2) with different concentrations of kanamycin and cultured at 28 °C and 200 rpm for 3 days before the photograph was taken; or method 2: the spore suspension was series diluted, spotted on SFM medium (2% soy flour, 2% mannitol, 2% agar) or fermentation medium (FM medium, formula as shown below) with 2% agar supplemented with different concentrations of kanamycin, and the plates were incubated at 30 °C for 6 days before the photograph was taken.

### 4.3. Fermentation, Extraction, and Quantitative Analysis of Elaiophylins

The cultivation of *Streptomyces* sp. 219807-derived strains and the analysis of the resulting products were as previously described [16]. The strains were cultured in liquid TSBY medium at 28 °C and 200 rpm for 3 days, respectively. Then 10% (*v*/*v*) of the culture was transferred in 50 mL of fresh fermentation medium (0.2% yeast extract, 1% glucose, 2.5% dextrin, 2% oatmeal, 1% cotton seed flour, 0.5% Fish meal, 0.3% CaCO_3_, pH 7.2) and fermented at 30 °C and 200 rpm for 8 days.

The mycelium and the culture supernatant were separated by centrifugation. The clarified supernatant after centrifugation was extracted with an equal volume of ethyl acetate three times. The ethyl acetate extracts were dried with anhydrous Na_2_SO_4_, filtered, and evaporated to dryness under reduced pressure. The mycelium was extracted with 30 mL of 80% (*v*/*v*) acetone water solution, which was combined with the culture supernatant extracts. The products were filtered through a 0.22 μm Nylon membrane before subjection to HPLC or LC-ESI-HRMS.

HPLC (Shimadzu, SPD-M20A/LC-20AT, Kyoto, Japan) and LC-ESI-HRMS (Thermo Scientific LTQ Orbitrap XL, positive ion mode, Waltham, MA, USA) were used to analyze samples. The HPLC conditions (Thermo Scientific C18 reversed-phase HPLC column, 250 × 4.6 mm, 5 μm; mobile phase A: water; mobile phase B: acetonitrile; UV detection λ: 252 nm) were as follows: elution gradient I (for HPLC): 10% B for 2 min, 10–100% B for 13 min, 100% B for 10 min, 100–10% B for 10 min, 10% B for 10 min at a flow rate of 1 mL/min; elution gradient II (for LC-ESI- HRMS): 10–100% B for 40 min, 100% B for 20 min, 100–10% B for 10 min, 10% B for 5 min at a flow rate of 0.5 mL/min.

The identity of resultant elaiophylin metabolites was confirmed by direct comparison of retention time and mass spectra with pure authentic standards. The production of elaiophylins was measured with the external standard law. The identities of azalomycin Fs and armeniaspirols were the same unless otherwise noted.

### 4.4. Fermentation, Extraction, and Quantitative Analysis of Azalomycin Fs

*Streptomyces* sp. 211726 and all derived strains were cultured in liquid TSBY medium at 30 °C with shaking at 200 rpm for 2 days, respectively. Then 10% (*v*/*v*) of the culture was transferred in 25 mL of fresh fermentation medium (0.2% yeast extract, 1% glucose, 3.5% soluble starch, 0.4% casein, pH 7.2–7.4) and fermented at 30 °C and 200 rpm for 10 days.

The mycelium and the culture supernatant were separated by centrifugation. The mycelium was extracted with 25 mL of methanol, which was combined with the clarified culture supernatant after centrifugation. The products were filtered through a microporous membrane (0.22 μm, nylon) before HPLC analysis.

Each sample was analyzed by HPLC (Shimadzu, SPD-M20A/LC 20AT, Kyoto, Japan) with a SHIMADZU Shim-pack VP-ODS C18 column (250 × 4.6 mm, 5 μm) at a flow rate of 1 mL/min using a mobile phase of (A) water and (B) methanol. The separation gradient was as follows: 70% B for 2 min, 70–90% B for 23 min, 90–70% B for 3 min, 70% B for 2 min. Azalomycin F was analyzed by LC-ESI-HRMS (Thermo Electron LTQ-ESI-HRMS, positive ion mode, Waltham, MA, USA) with a SHIMADZU Shim-pack VP-ODS C18 column (250 × 4.6 mm, 5 μm) at a flow rate of 0.2 mL/min using a mobile phase of (A) H_2_O with 0.1% formic acid and (B) methanol. The separation gradient was as follows: 0–2 min, 80% B; 2–25 min, 80–100% B; 25–28 min, 100–80% B; 28–43 min, 80% B. The mass spectrometer was set to full scan (from 200 to 2000 m/z).

### 4.5. Fermentation, Extraction, and Quantitative Analysis of Armeniaspirols

The cultivation of *S. armeniacus* strains and the analysis of the resulting products were as previously described [4]. The *S. armeniacus* strains were cultured in liquid ISP-2 medium (0.4% yeast extract, 1% malt extract, 0.4% glucose, pH 7.0) containing 2 g/L CaCO_3_ at 30 °C and 200 rpm for 3 days, respectively. Then 10% (*v*/*v*) of the culture was transferred in 50 mL of fresh ISP-2 medium and fermented at 30 °C and 200 rpm for 6 days.

The culture was separated from the biomass fraction and the supernatant fraction by centrifugation. The supernatant fraction was extracted with an equal volume of ethyl acetate twice. The biomass fraction was extracted with methanol, disrupted by sonication, and centrifuged to remove the cell debris. The retained supernatant was evaporated under reduced pressure, resuspended in water, and twice extracted with an equal volume of ethyl acetate. The combined organic extracts were dried over anhydrous Na_2_SO_4_, filtered, and concentrated on a rotovap. The residue was re-dissolved in methanol and filtered with a microporous membrane (0.22 μm, nylon).

HPLC (Shimadzu, SPD-M20A/LC-20AT, Kyoto, Japan) and LC-ESI-HRMS (Thermo Scientific LTQ Orbitrap XL, negative ion mode, Waltham, MA, USA) were used to analyze each sample. The HPLC conditions (mobile phase A: water; mobile phase B: acetonitrile; UV detection λ: 300 nm) were as follows: elution gradient I (for Thermo Scientific C18 reversed-phase HPLC column, 250 × 4.6 mm, 5 μm, used in HPLC and LC-ESI-HRMS): 5–95% B for 25 min, 95% B for 10 min, 95–5% B for 5 min, 5% B for 5 min at a flow rate of 1 mL/min.

## 5. Conclusions

Four strong constitutive promoters, *kasO*p*, *hrdB*p, *SCO5768*p, and SP44, were inserted in the integrative vector pSET152 to generate four new vectors, and three of them, pWHU1288 with *hrdB*p, pWHU1290 with *kasO*p*, and pWHU1291 with SP44, were proven to be functional in different host strains of six *Streptomyces* species, namely *S. coelicolor* M145, *S. lividans* TK24, *S. olivaceus* CGMCC 4.1369, *Streptomyces* sp. 219807, *Streptomyces* sp. 211726, and *S. armeniacus* DSM 43125. Furthermore, pWHU1288 was selected to overexpress NAD(P)-dependent oxidoreductase gene ela8* in marine *Streptomyces* sp. 219807 and the elaiophylin production increased about 108%. Similarly, pWHU1288 harboring the 4-guanidinobutyryl-CoA ligase gene *azl4* enhanced the elaiophylin production of marine *Streptomyces* sp. 211726 about 136%. In addition, pWHU1290 was used to overexpress the PKS gene arm6 in *S. armeniacus* DSM 43125 and armeniaspirol production improved about 114%. In brief, pWHU1288 and pWHU1290 exhibit efficient gene activation and expression, and flexible host compatibility, which are useful in synthetic biology.

## Figures and Tables

**Table 1:** Kanamycin resistance levels conferred by different promoters in different *Streptomyces* strains.

Strain	Medium	Kanamycin Resistance Levels (μg/mL)					
		None	*ermE*p*	*hrdB*p	*SCO5768*p	*kasO*p*	SP44
*S. coelicolor* M145	SFM ^1^	0	200	400	0	200	800–1000
*S. lividans* TK24	SFM	0	200	200	1000	400–800	1500–2000
*S. olivaceus* CGMCC 4.1369	SFM	0–50	400	400	400	400	1000
*Streptomyces* sp. 219807	FM ^2^	0	200	800–1000	0	800–1000	1000–1200
*Streptomyces* sp. 211726	FM	0	100–200	300–400	100–200	200	600
*S. armeniacus* DSM 43125	FM	0	900	900	2200	2200	1800

^1^ SFM, Soy Flour Mannitol medium; ^2^ FM, fermentation medium.

## Data Availability

The authors declare that all data of this study are available within the article and its Supplementary Materials file or from the corresponding authors upon request.

## Supplementary Materials

The following supporting information can be downloaded at: https://www.mdpi.com/article/10.3390/md22020094/s1, Table S1: Bacterial strains and plasmids used in this study; Table S2: Primers used in this study; Figures S1–S2: Agarose gel electrophoresis analysis of PCR products or recombinant plasmids digested with restriction enzymes; Figures S3–S10: Determination of promoter activity by using kanamycin resistance gene *neo* as a reporter gene; Table S3: Deduced functions and sequence comparison of elaiophylin biosynthetic genes; Figure S11: Proposed biosynthetic pathway of elaiophylin; Figure S12: Relative levels of elaiophylin (and azalomycin F) production by *Streptomyces* sp. 219807 (and 211726) derivative strains detected and quantified by HPLC.
