# Supplementary material for: Development of Integrated Vectors with Strong Constitutive Promoters for High-Yield Antibiotic Production in Mangrove-Derived Streptomyces
Source: Mar Drugs. 2024 Feb 18;22(2):94. doi: 10.3390/md22020094 (PMC10890193; doi:10.3390/md22020094)
Supplement: Supplementary file 1 [file marinedrugs-22-00094-s001.zip › marinedrugs-2875846-supplementary.pdf]

Development of Integrated Vectors with Strong Constitutive Promoters for High-Yield Antibiotic Production in Mangrove-Derived *Streptomyces*

Mingxia Zhao †, Zhiqiang Yang †, Xinyue Li, Yaqi Liu, Yingying Zhang, Mengqian Zhang, Yangli Li, Xincheng Wang, Zixin Deng, Kui Hong * and Dongqing Zhu *

Key Laboratory of Combinatorial Biosynthesis and Drug Discovery, Ministry of Education, School of Pharmaceutical Sciences, Wuhan University, Wuhan 430071, China

* Correspondence: kuihong31@whu.edu.cn (K.H.); dzhu2011@whu.edu.cn (D.Z.)

† These authors contributed equally to this work.

Table S1**.** Bacterial strains and plasmids used in this study

**Strain or plasmid**	**Relevant phenotype and/or characteristics**	**Source or reference**
Streptomyces strain		
S. coelicolor M145	Wild-type strain,	
S. lividans TK24	Wild-type strain,	
S. venezuelae ISP 5230	Wild-type strain,	
S. olivaceus CGMCC 4.1369	Wild-type strain	CGMCC
S. armeniacus DSM 43125	Wild-type strain, streptopyrrole and armeniaspirol producer	DSMZ, [1]
Streptomyces sp. 219807	Wild-type strain, elaiophylin producer	[2]
Streptomyces sp. 211726	Wild-type strain, azalomycin F producer	[3, 4]
Escherichia coli strain		
DH10B	F^- *mcrA, Δ(mrr-hsdRMS-mcrBC)*, φ80d*lacZΔM15, ΔlacX74, recA1, endA1, araD139, Δ(ara, leu)7697, galU, galK, rpsL, nupG*	Gibco BRL
ET12567/pUZ8002	F^- *ara-14, leuB6, fhuA13, lacY1, tsx-78, supE44, glnV44, galK2, galT22, mcrA, dcm-6, hisG4, rfbD1, rpsL136, dam-13::*Tn*9, xyl-5, mtl-1, recF143, thi-1, mcrB, hsdR2, hsdS::*Tn*10* pUZ8002*:* (derivative of pUB307, *tra*)	[5, 6]
Plasmid		
pEASY-BLUNT Zero vector	*bla*, *lacZα*, T7 promoter	TransGen
pESI-Blunt simple vector	*bla*, *neo*, *lacZα*, T7 promoter,	Yeasen
pHZ1358	pIJ101 derivative, *bla*, *tsr*, *oriT*, *sti*	[7]
pSET152	*aac(3)IV, oriT, int*	[8]
pIB139	*aac(3)IV*, *oriT*, *int*, *ermE*p*	[9]
pIB-KasOp*	*aac(3)IV*, *oriT*, *int*, *kasO*p*	Group Xudong Qu [10]
pWHU2449	*aac(3)IV*, *oriT*, *int*, *ermE*p*-*sfp*-s*vp*	[11]
pJTU3957	*aac(3)IV*, *oriT*, *int*, *ermE*p*-*adpA*-*vgb*	Group Delin You [12]
pSET152::P_{hrdB}*G*	*aac(3)IV*, *oriT*, *int*, *hrdB*p-*sanG*	Group Huarong Tan [13]
pWHU1288	**pSET152-*hrdB*p**. 428-bp XbaI + BamHI DNA fragment carrying the promoter of *hrdB* amplified from pSET152::P_{hrdB}*G* by using primer pair hrdB-pF-XbaI and hrdB-pR-NdeI-BamHI, inserted into the corresponding site of pSET152	This work
pWHU1289	**pSET152-*SCO5768*p.** 287-bp XbaI + BamHI DNA fragment carrying the promoter of SCO5768 amplified from pLXY35 by using primer pair DQ200F and DQ200R, inserted into the corresponding site of pSET152	This work

pWHU1290	**pSET152-*kasO*p***. 113-bp XbaI + BamHI DNA fragment carrying the promoter of *kasO* amplified from pIB-*KasO*p* by using primer pair DQ222F and DQ222R, inserted into the corresponding site of pSET152	This work
pWHU1291	**pSET152-SP44.** 428-bp XbaI + NdeI DNA fragment carrying the promoter of *hrdB* of pWHU1288 replaced by 150-bp XbaI + NdeI DNA fragment carrying the promoter of SP44 synthetized directly	This work
pWHU1292	**pSET152-*neo***. 809-bp DNA fragment carrying *neo* gene amplified from pHZ1358 by using primer pair DQ195F and DQ195R, digested with EcoRI, inserted into EcoRV+EcoRI site of pSET152	This work
pWXC4	**pSET152-SP44-*neo*.** 795-bp NdeI + EcoRI DNA fragment carrying *neo* recovered form pLXY36, inserted into the corresponding site of pWHU1291	This work
pLXY20	2121-bp DNA fragment carrying the promoter of *ela2** and *ela3** amplified from the DNA of *Streptomyces* sp. 219807 by using primer pair DQ187F and DQ187R, inserted into pEasy-Blunt Zero Vector	This work
pLXY32	2274-bp DNA fragment carrying the promoter of *ela2* and *ela1* amplified from the DNA of *Streptomyces* sp. 219807 by using primer pair DQ196F and DQ196R, inserted into pEasy-Blunt Zero Vector	This work
pLXY33	1798-bp DNA fragment carrying the promoter of *ela4** and *ela5** amplified from the DNA of *Streptomyces* sp. 219807 by using primer pair DQ197F and DQ197R, inserted into pEasy-Blunt Zero Vector	This work
pLXY34	2237-bp DNA fragment carrying the promoter of *ela9** and *ela10**amplified from the DNA of *Streptomyces* sp. 219807 by using primer pair DQ199F and DQ199R, inserted into pEasy-Blunt Zero Vector	This work
pLXY35	463-bp DNA fragment carrying the promoter of SCO5768 amplified from the DNA of *S. coelicolor* M145 by using primer pair DQ188F and DQ188R, inserted into pEasy-Blunt Zero Vector	This work
pLXY36	809-bp DNA fragment carrying *neo* gene amplified from pHZ1358 by using primer pair DQ195F and DQ195R, inserted into pEasy-Blunt Zero Vector	This work
pLXY37	**pIB139-*neo***. 795-bp NdeI + EcoRI DNA fragment carrying *neo* recovered form pLXY36, inserted into the corresponding site of pIB139	This work
pLXY39	**pSET152-*hrdB*p-*neo***. 795-bp NdeI + EcoRI DNA fragment carrying *neo* recovered form pLXY36, inserted into the corresponding site of pWHU1288	This work
pLXY40	**pSET152-*SCO5768*p-*neo***. 795-bp NdeI + EcoRI DNA fragment carrying *neo* recovered form pLXY36, inserted into the corresponding site of pWHU1289	This work
pLXY41	**pSET152-*kasO*p*-*neo***. 795-bp NdeI + EcoRI DNA fragment carrying *neo* recovered form pLXY36, inserted into the corresponding site of pWHU1290	This work

pLXY44	**pSET152-*hrdB*p-*ela2**-*ela3**.** 1878-bp NdeI + EcoRI DNA fragment carrying *ela2** and *ela3** amplified from pLXY20 by using primer pair LXY33F and LXY33R, inserted into the corresponding site of pWHU1288	This work
pLXY45	**pSET152-*hrdB*p-*ela2*-*ela1***. 1865-bp NdeI + PmeI DNA fragment carrying *ela2** and *ela3** of pLXY44 replaced by 1939-bp NdeI + PmeI DNA fragment carrying *ela2* and *ela1* amplified from pLXY32 by using primer pair LXY34F and LXY34R	This work
pLXY46	**pSET152-*hrdB*p-*ela7**.** 1865-bp NdeI + PmeI DNA fragment carrying *ela2** and *ela3** of pLXY44 replaced by 669-bp NdeI + PmeI DNA fragment carrying *ela7** amplified from the DNA of *Streptomyces* sp. 219807 by using primer pair LXY41F and LXY41R, and primer pair LXY43F and LXY43R (nested PCR)	This work
pLXY47	**pSET152-*hrdB*p- *ela4**-*ela5****. 1865-bp NdeI + PmeI DNA fragment carrying *ela2** and *ela3** of pLXY44 replaced by 1675-bp NdeI + PmeI DNA fragment carrying *ela4** and *ela5** amplified from pLXY33 by using primer pair LXY35F and LXY35R	This work
pLXY48	**pSET152-*hrdB*p-*ela8****. 1865-bp NdeI + PmeI DNA fragment carrying *ela2** and *ela3** of pLXY44 replaced by 966-bp NdeI + PmeI DNA fragment carrying *ela8** amplified from the DNA of *Streptomyces* sp. 219807 by using primer pair LXY30F and LXY30R, and primer pair LXY36F and LXY36R (nested PCR)	This work
pLXY49	**pSET152-*hrdB*p-*ela10****. 1865-bp NdeI + PmeI DNA fragment carrying *ela2** and *ela3** of pLXY44 replaced by 1410-bp NdeI + PmeI DNA fragment carrying *ela10** amplified from pLXY34 by using primer pair LXY38F and LXY38R	This work
pLXY50	**pSET152-*hrdB*p-*ela9****. 1865-bp NdeI + PmeI DNA fragment carrying *ela2** and *ela3** of pLXY44 replaced by 667-bp NdeI + PmeI DNA fragment carrying partial *ela9** recovered form pLXY53 and 331-bp FspI + PmeI fragment carrying partial *ela9** recovered form pLXY54	This work
pLXY51	**pSET152-*hrdB*p-*ela3***. 1865-bp NdeI + PmeI DNA fragment carrying *ela2** and *ela3** of pLXY44 replaced by 2877-bp NdeI + PmeI DNA fragment carrying *ela3* amplified from the DNA of *Streptomyces* sp. 219807 by using primer pair LXY39F and LXY39R, and primer pair LXY40F and LXY40R (nested PCR)	This work
pLXY52	**pSET152-*hrdB*p-*ela6****. 1865-bp NdeI + PmeI DNA fragment carrying *ela2** and *ela3** of pLXY44 replaced by 1263-bp NdeI + PmeI DNA fragment carrying *ela6** amplified from the DNA of *Streptomyces* sp. 219807 by using primer pair LXY41F and LXY41R, and primer pair LXY42F and LXY42R (nested PCR)	This work
pLXY53	772-bp DNA fragment carrying partial *ela9** amplified from the DNA of *Streptomyces* sp. 219807 by using primer pair LXY30F and LXY30R, and primer pair LXY37F and LXY37R (nested PCR), inserted into pEasy-Blunt Zero Vector	This work

pLXY54	573-bp DNA fragment carrying partial *ela9** amplified from the DNA of *Streptomyces* sp. 219807 by using primer pair DQ199F and LXY37R, inserted into pEasy-Blunt Zero Vector	This work
pLXY55	**pSET152-*hrdB*p-*adpA*.** 1865-bp NdeI + PmeI DNA fragment carrying *ela2** and *ela3** of pLXY44 replaced by 1229-bp NdeI + PmeI DNA fragment carrying *adpA* amplified from pJTU3957 by using primer pair adpA-F and adpA-R	This work
pLXY56	**pSET152-*hrdB*p-*vgb***. 1865-bp NdeI + PmeI DNA fragment carrying *ela2** and *ela3** of pLXY44 replaced by 488-bp NdeI + PmeI DNA fragment carrying *vgb* amplified from pJTU3957 by using primer pair vgb-F and vgb-R	This work
pLXY61	5004-bp DNA fragment carrying *elaB* amplified from the DNA of *Streptomyces* sp. 219807 by using primer pair LXY44F and LXY44R, inserted into pEasy-Blunt Zero Vector	This work
pLXY64	792-bp DNA fragment carrying *ela1**amplified from the DNA of *Streptomyces* sp. 219807 by using primer pair LXY48F and LXY48R, and primer pair LXY49F and LXY49R (nested PCR), inserted into pEasy-Blunt Zero Vector	This work
pDQ137	**pSET152-*hrdB*p-*ela1****.792-bp NdeI + PmeI DNA fragment carrying *ela1** recovered from pLXY64, inserted into NdeI+EcoRV site of pWHU1288	This work
pDQ138	4947-bp DNA fragment carrying *elaC* amplified from the DNA of *Streptomyces* sp. 219807 by using primer pair LXY45F and LXY45R, inserted into pEasy-Blunt Zero Vector	This work
pDQ139	**pSET152-*hrdB*p-*elaC***. 4947-bp NdeI + PmeI DNA fragment carrying *elaC* recovered from pDQ138, inserted into NdeI+EcoRV site of pWHU1288	This work
pNN1	**pSET152-*hrdB*p-*elaB***. 1865-bp NdeI + PmeI DNA fragment carrying *ela2** and *ela3** of pLXY44 replaced by 5004-bp NdeI + PmeI DNA fragment carrying *elaB* recovered from pLXY61	This work
pMX5	1467-bp NdeI + EcoRI DNA fragment carrying *azl4* amplified from the DNA of *Streptomyces* sp. 211726 by using primer pairDQ201F and DQ201R, and primer pair MX01F and MX01R (nested PCR), inserted into the corresponding site of pIB139	This work
pMX6	1058-bp NdeI + EcoRI DNA fragment carrying *azl5* amplified from the DNA of *Streptomyces* sp. 211726 by using primer pairDQ201F and DQ201R, and primer pair MX02F and MX02R (nested PCR), inserted into the corresponding site of pIB139	This work
pMX7	404-bp NdeI + EcoRI DNA fragment carrying *azl8* amplified from the DNA of *Streptomyces* sp. 211726 by using primer pairDQ203F and DQ203R, and primer pair MX04F and MX04R (nested PCR), inserted into the corresponding site of pIB139	This work
pMX8	812-bp NdeI + EcoRI DNA fragment carrying *azl10* amplified from the DNA of *Streptomyces* sp. 211726 by using primer pairDQ204F and DQ204R, and primer pair MX05F and MX05R (nested PCR), inserted into the corresponding site of pIB139	This work

pMX10	829-bp NdeI + EcoRI DNA fragment carrying *azl13* amplified from the DNA of *Streptomyces* sp. 211726 by using primer pairDQ207F and DQ207R, and primer pair MX08F and MX08R (nested PCR), inserted into the corresponding site of pIB139	This work
pMX11	1470-bp NdeI + EcoRI DNA fragment carrying *azl14* amplified from the DNA of *Streptomyces* sp. 211726 by using primer pair DQ207F and DQ207R, and primer pair MX09F and MX09R (nested PCR), inserted into the corresponding site of pIB139	This work
pMX12	827-bp DNA fragment carrying *azl12* amplified from the DNA of *Streptomyces* sp. 211726 by using primer pairDQ206F and DQ206R, and primer pair MX07F and MX07R (nested PCR), inserted into pEasy-Blunt Zero Vector	This work
pMX13	633-bp DNA fragment carrying *azl6* amplified from the DNA of *Streptomyces* sp. 211726 by using primer pairDQ202F and DQ202R, and primer pair MX03F and MX03R (nested PCR), inserted into pEasy-Blunt Zero Vector	This work
pMX16	1308-bp DNA fragment carrying *azl11* amplified from the DNA of *Streptomyces* sp. 211726 by using primer pairDQ205F and DQ205R, and primer pair MX06F and MX06R2 (nested PCR), inserted into pEasy-Blunt Zero Vector。	This work
pMX301	**pSET152-*hrdB*p-*azl4***. 1467-bp NdeI + EcoRI DNA fragment carrying *azl4* recovered from pMX5, inserted into the corresponding site of pWHU1288	This work
pMX302	**pSET152-*hrdB*p-*azl5***. 1058-bp NdeI + EcoRI DNA fragment carrying *azl5* recovered from pMX6, inserted into the corresponding site of pWHU1288	This work
pMX303	**pSET152-*hrdB*p-*azl6***. 633-bp NdeI + EcoRI DNA fragment carrying *azl6* recovered from pMX13, inserted into the corresponding site of pWHU1288	This work
pMX304	**pSET152-*hrdB*p-*azl8***. 404-bp NdeI + EcoRI DNA fragment carrying *azl8* recovered from pMX7, inserted into the corresponding site of pWHU1288	This work
pMX305	**pSET152-*hrdB*p-*azl10***. 812-bp NdeI + EcoRI DNA fragment carrying *azl10* recovered from pMX8, inserted into the corresponding site of pWHU1288	This work
pMX306	**pSET152-*hrdB*p-*azl11***. 1308-bp NdeI + EcoRI DNA fragment carrying *azl11* recovered from pMX16, inserted into the corresponding site of pWHU1288	This work
pMX307	**pSET152-*hrdB*p-*azl12***. 827-bp NdeI + EcoRI DNA fragment carrying *azl12* recovered from pMX12, inserted into the corresponding site of pWHU1288	This work
pMX308	**pSET152-*hrdB*p-*azl13***. 829-bp NdeI + EcoRI DNA fragment carrying *azl13* recovered from pMX10, inserted into the corresponding site of pWHU1288	This work
pMX309	**pSET152-*hrdB*p-*azl14***. 1470-bp NdeI + EcoRI DNA fragment carrying *azl14* recovered from pMX11, inserted into the corresponding site of pWHU1288	This work

pMX401	**pSET152-SP44-*ela8*****. 966-bp NdeI + EcoRI DNA fragment carrying *ela8** recovered from pLXY48, inserted into the corresponding site of pWHU1291	
pMX402	2877-bp DNA fragment carrying *ela3* amplified from the DNA of *Streptomyces* sp. 219807 by using primer pair LXY39F and LXY39R, and primer pair LXY40F and orf8003R (nested PCR), inserted into pESI-Blunt Zero Vector	
pMX403	**pSET152-SP44-*azl6***. 633-bp NdeI + EcoRI DNA fragment carrying *azl6* recovered from pMX13, inserted into the corresponding site of pWHU1291	
pMX404	**pSET152-SP44-*azl4***. 1467-bp NdeI + EcoRI DNA fragment carrying *azl4* recovered from pMX301, inserted into the corresponding site of pWHU1291	
pMX405	**pSET152-SP44-*ela3*****.2877-bp NdeI + EcoRV DNA fragment carrying *ela3* recovered from pMX402, inserted into the corresponding site of pWHU1291	
pYQ1	**pSET152-*kasO*p*-*arm1*.** 1281-bp NdeI + EcoRI DNA fragment carrying *arm1* amplified from the DNA of *S. armeniacus* DSM 43125 by using primer pair LY2F and LY2R, inserted into the corresponding site of pWHU1290	This work
pYQ2	**pSET152-*kasO*p*-*arm24*.** 891-bp NdeI + EcoRI DNA fragment carrying *arm24* amplified from the DNA of *S. armeniacus* DSM 43125 by using primer pair LY3F and LY3R, inserted into the corresponding site of pWHU1290	This work
pYQ3	**pSET152-*kasO*p*-*arm25*.** 615-bp BamHI + EcoRI DNA fragment carrying *arm25* amplified from the DNA of *S. armeniacus* DSM 43125 by using primer pair LY4F and LY4R, inserted into the corresponding site of pWHU1290	This work
pZQ5	4505-bp DNA fragment carrying part of *arm6* amplified from the DNA of *S. armeniacus* DSM 43125 by using primer pair ZQ5F and ZQ5R, inserted into pESI-Blunt simple vector	This work
pZQ6	4956-bp DNA fragment carrying part of *arm6* amplified from the DNA of *S. armeniacus* DSM 43125 by using primer pair ZQ6F and ZQ6R, inserted into pESI-Blunt simple vector	This work
pZQ7	4397-bp DNA fragment carrying *arm7* amplified from the DNA of *S. armeniacus* DSM 43125 by using primer pair ZQ7F and ZQ7R, inserted into pESI-Blunt simple vector	This work
pZQ11	**pSET152-*kasO*p*-*arm6*.** 4220-bp NdeI + FspI DNA fragment carrying part of *arm6* recovered from pZQ5 and 4684-bp FspI + EcoRI DNA fragment carrying part of *arm6* recovered from pZQ6, inserted into the corresponding site of pWHU1290	This work
pZQ12	**pSET152-*kasO*p*-*arm7*.** 4398-bp NdeI + EcoRI DNA fragment carrying *arm7* recovered from pZQ7, inserted into the corresponding site of pWHU1290	This work

Table S2. Primers used in this study

Primer name	Sequence (5'-3'), (restriction enzyme site underlined)	Purpose
adpA-F	GGCTTAGCCATATGAGCCAC (NdeI)	*adpA*
adpA-R	GTGGCCGTTTAAACGACTAGTTCACGGCGCGC (PmeI, SpeI)	*adpA*
DQ187F	CCCGACGACCCATCGACTGA	*ela2* + ela3**
DQ187R	CGTGATTGCGGTATTCCTTGC	*ela2* + ela3**
DQ188F	ATGTAGTCCCGCAACAACG	*SCO5768*p
DQ188R	CCGAATTGCCGTTCAATCTGT	*SCO5768*p
DQ195F	GTTTCATATGATTGAACAAGATGGA (NdeI)	*neo*
DQ195R	AGTGAATTCAGAAGAACTCGTCAAG (EcoRI)	*neo*
DQ196F	CCAGCCTGTTCATCAACATCA	*ela2 + ela1*
DQ196R	GGGAGTTCTCCGACATCACGA	*ela2 + ela1*
DQ197F	AGAAACGAGCGAGGAGGAAC	*ela4* + ela5**
DQ197R	GCCGTGGACAGAAACAGCAGA	*ela4* + ela5**
DQ199F	CAGGCGATGGAGACGCTGAC	*ela9* + ela10**
DQ199R	GGGTCCTTGTCCTTGGTGGC	*ela9* + ela10**
DQ200F	GATGGATCC CATATGCGTCCCCT (BamHI, NdeI)	*SCO5768*p
DQ200R	TTCCTCTAGAGGGTTCCAACCGGT (XbaI)	*SCO5768*p
DQ201F	AAGAAAGGCTCAGTGAAACCCGA	*azl 4 + azl 5*
DQ201R	CCTTCCTCCGTGCCATACCG	*azl 4 + azl 5*
DQ202F	CGCACCGTGGACAACTTCG	*azl 6*
DQ202R	AGGGTTTCGGGACTGAATGGT	*azl 6*
DQ203F	CTTGTGGTGACTCGGGAACTG	*azl 8*
DQ203R	TGGCGAAGCTGACCGTCTA	*azl 8*
DQ204F	CGGCTACAGTGCGAGGGAT	*azl 10*
DQ204R	GGAGCACGACGAGTGGAAGA	*azl 10*
DQ205F	ACTTCTTCCACTCGTCGTGCTCC	*azl 11*
DQ205R	TGTCCGTGGCCGAATCTGTG	*azl 11*
DQ206F	CCATGCGATCTGTGGAGTCAGC	*azl 12*
DQ206R	CAGTGCCTTCGACAGTGGGTGT	*azl 12*
DQ207F	GTTCCTGGGTCGCCATTCC	*azl 13+ azl 14*
DQ207R	CCGTCCAACCCGTACCAGAG	*azl 13+ azl 14*
DQ222F	TCTAGTTCTAGATGTTCACATTCGA (XbaI)	*kasO*p*
DQ222R	TAGAGGATCCCCAACATATGAACTC (BamHI, NdeI)	*kasO*p*

hrdB-pF-XbaI	AATTTCTAGAACGCCTTCCGCCGGAACG (XbaI)	*hrdB*p
hrdB-pR-NdeI-BamHI	AATTGGATCC CATATGCAAACTCTCGGAACGTTG (BamHI, NdeI)	*hrdB*p
LXY30F	GCCAATACATATGCCAAGTGCTCAGGCTTATCG (NdeI)	*ela8**
LXY30R	CAAGGTACCCGTCGCACAGCTTCTCGTAC (KpnI)	*ela8**
LXY33F	GGAATTCCATATGCGCATCCTTTTCGCG (NdeI)	*ela2** + *ela3**
LXY33R	GGGAATTCGTGGCCGTTTAAACGACTAGTTCAGCCGCGCCGGGCG (EcoRI, PmeI, SpeI)	*ela2** + *ela3**
LXY34F	GGAATTCCATATGCCCAACCCTGATTAACC (NdeI)	*ela2* + *ela1*
LXY34R	GTGGCCGTTTAAACGACTAGTTCACATCTTCTTGAGCGGTT (PmeI, SpeI)	*ela2* + *ela1*
LXY35F	GGAATTCCATATGCCGCTGATCGAGGTCAG (NdeI)	*ela4** + *ela5**
LXY35R	GTGGCCGTTTAAACGACTAGTTCACTCCCAGCGGAACAGCT (PmeI, SpeI)	*ela4** + *ela5**
LXY36F	GGAATTCCATATGGGCCACATCCGAGATCG (NdeI)	*ela8**
LXY36R	GTGGCCGTTTAAACGACTAGTTCAGTGTCCTTCGGTACGGG (PmeI, SpeI)	*ela8**
LXY37F	GGAATTCCATATGCAGTACAACTATCTCGG (NdeI)	*ela9**
LXY37R	GTGGCCGTTTAAACGACTAGTTCAACACGCCCACGCCTCCG (PmeI, SpeI)	*ela9**
LXY38F	GGAATTCCATATGGTGGTGAGCACGCTTCAGGA (NdeI)	*ela10**
LXY38R	GTGGCCGTTTAAACGACTAGTCTATACGGCCAGGGAGTGCA (PmeI, SpeI)	*ela10**
LXY39F	TCCGCTTTATGGCATGAGAA	*ela3*
LXY39R	GGTTTGTTGTAGACGGGAAGG	*ela3*
LXY40F	GGAATTCCATATGGTGTTTTCATCGGCCAG (NdeI)	*ela3*
LXY40R	GTGGCCGTTTAAACGACTAGTTCAGGCGATTTCGTCCAACT (PmeI, SpeI)	*ela3*
LXY41F	CCGCCTTCGACAACAAAC	*ela6** + *ela7**
LXY41R	CAACGAGCCCAGATGAAAC	*ela6** + *ela7**
LXY42F	GGAATTCCATATGGTGAGCGCGTCCAAGGACGA (NdeI)	*ela6**
LXY42R	GTGGCCGTTTAAACGACTAGTTCATGCAACGGCTTCCTGGG (PmeI, SpeI)	*ela6**
LXY43F	GGAATTCCATATGAGCGGAAACAACCATGG (NdeI)	*ela7**
LXY43R	GTGGCCGTTTAAACGACTAGTTCATTCGAATAGATGTCGTT (PmeI, SpeI)	*ela7**
LXY44F	GGGCTTCCATATGGCGAACGAGAACGAA (NdeI)	*elaB*
LXY44R	CCGGGGGTTTAAACGACTAGTTCATGGGGCCGTCAGATC (PmeI, SpeI)	*elaB*
LXY45F	GCTGACGCATATGGACAACGAGAAGAAG (NdeI)	*elaC*
LXY45R	CAGCTCGTTTAAACGACTAGTTCAGAGGTCGTTCTTGTC (PmeI, SpeI)	*elaC*
LXY48F	CCCCGCATCAGATAAGGAA	*ela1**
LXY48R	GGTCTGGTACGCGAACATCA	*ela1**
LXY49F	TAAGGACATATGACCATGGCGTTGCCC (NdeI)	*ela1**
LXY49R	TGCGCAGTTTAAACGACTAGTTCAGTCGATGGGTCGTCG (PmeI, SpeI)	*ela1**

LY2F	CGGAATTCACTAGTTCACATGGCCTGGGACTC（EcoRI，SpeI）	*arm1*
LY2R	GGAATTCCATATGACAGGCAGTCCGTGGA（NdeI）	*arm1*
LY3F	CGGAATTCACTAGTTCAGGCGTGCGACCG（EcoRI，SpeI）	*arm24*
LY3R	GGAATTCCATATGGTGGAGATCAATGTCCTGGGG（NdeI）	*arm24*
LY4F	CGGAATTCACTAGTTCAGGCAAGCCATCCGGCC（EcoRI，SpeI）	*arm25*
LY4R	CGGGATCCATGCCCGAACTGAGGGTGC（BamHI）	*arm25*
MX01F	GTGAAACCATATGACCGCAAAGGTCTTT (NdeI)	*azl 4*
MX01R	CTGAATTCTCTAGAGGTCCGTCATGCCTT (EcoRI)	*azl 4*
MX02F	GAGCGAACATATGACGGACCCGCAGAACA (NdeI)	*azl 5*
MX02R	GGAATTCTCTAGATCACCGCCTCGCCCG (EcoRI)	*azl 5*
MX03F	GCGTGCCCATATGGCACGGAGGAAGGAG (NdeI)	*azl 6*
MX03R	GCGAATTCTCTAGATCAGTCGGTGACGGT (EcoRI)	*azl 6*
MX04F	GAGTGACCATATGGGGACCAAGCAGTAC (NdeI)	*azl 8*
MX04R	AGAATTCTCTAGATCAGGCGTCGAAGCGG (EcoRI)	*azl 8*
MX05F	AGGATCTCATATGACCTCCACACCACAC (NdeI)	*azl 10*
MX05R	CGAATTCTCTAGATCAGCAGCCGATGCG (EcoRI)	*azl 10*
MX06F	CCGGAGCCATATGGTGACCACCGCCCC (NdeI)	*azl 11*
MX06R2	CCGAATTCACTAGTCGTCAGCAGACGCC (EcoRI)	*azl 11*
MX07F	CCGTACGCATATGGTGCGGAGGGC (NdeI)	*azl 12*
MX07R	CGAATTCTCTAGATCAGCCGTCCTCGC (EcoRI)	*azl 12*
MX08F	CGAAGGCACATATGAAGATCTCCGGACT (NdeI)	*azl 13*
MX08R	CAGAATTCTCTAGATCAGCATGACGGGTC (EcoRI)	*azl 13*
MX09F	AAGGACCCCATATGCTGACCGACCC (NdeI)	*azl 14*
MX09R	CGAATTCTCTAGACATTGGGTCTCACCC (EcoRI)	*azl 14*
Orf8003R	CGTGATATCTCAGGCGATTTCGTCCAACT (EcoRV)	*ela3*
vgb-F	GGAGAACCATATGCTGGAAC (NdeI)	*vgb*
vgb-R	GTGGCCGTTTAAACGACTAGTTCACTCGAAC (PmeI, SpeI)	*vgb*
ZQ5F	GGACATATGGTCTCCAGTGACG (NdeI)	*arm6*
ZQ5R	CACGGGATGTCAGGGCTC	*arm6*
ZQ6F	CGGCGTCAACCTCATCCTCAG	*arm6*
ZQ6R	TCGAATTCAGCGCCTGTACGAG (EcoRI)	*arm6*
ZQ7F	GGACATATGACGTCGCCGGTAC (NdeI)	*arm7*
ZQ7R	CGCGAATTCATCGGTTACCT (EcoRI)	*arm7*

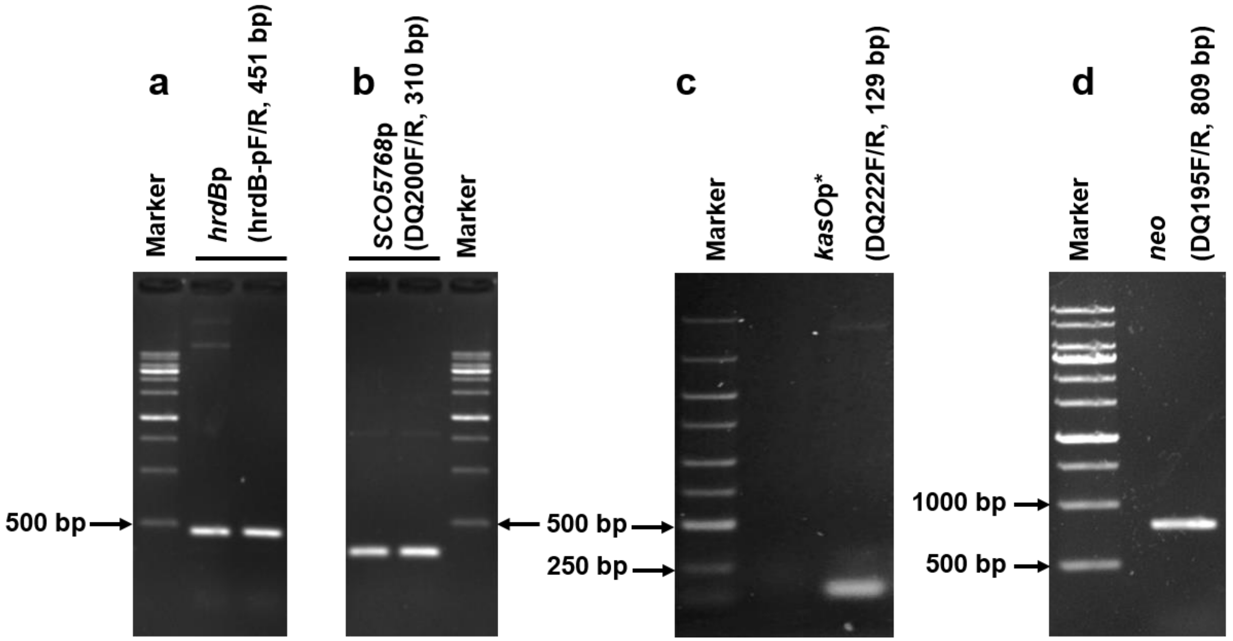

Figure S1 Agarose gel electrophoresis analysis of PCR products. Gels were stained with ethidium bromide and photographed under UV light at 305 nm. a, *hrdB*p; b, *SCO5768*p; c, *kasO*p*; d, *neo*.

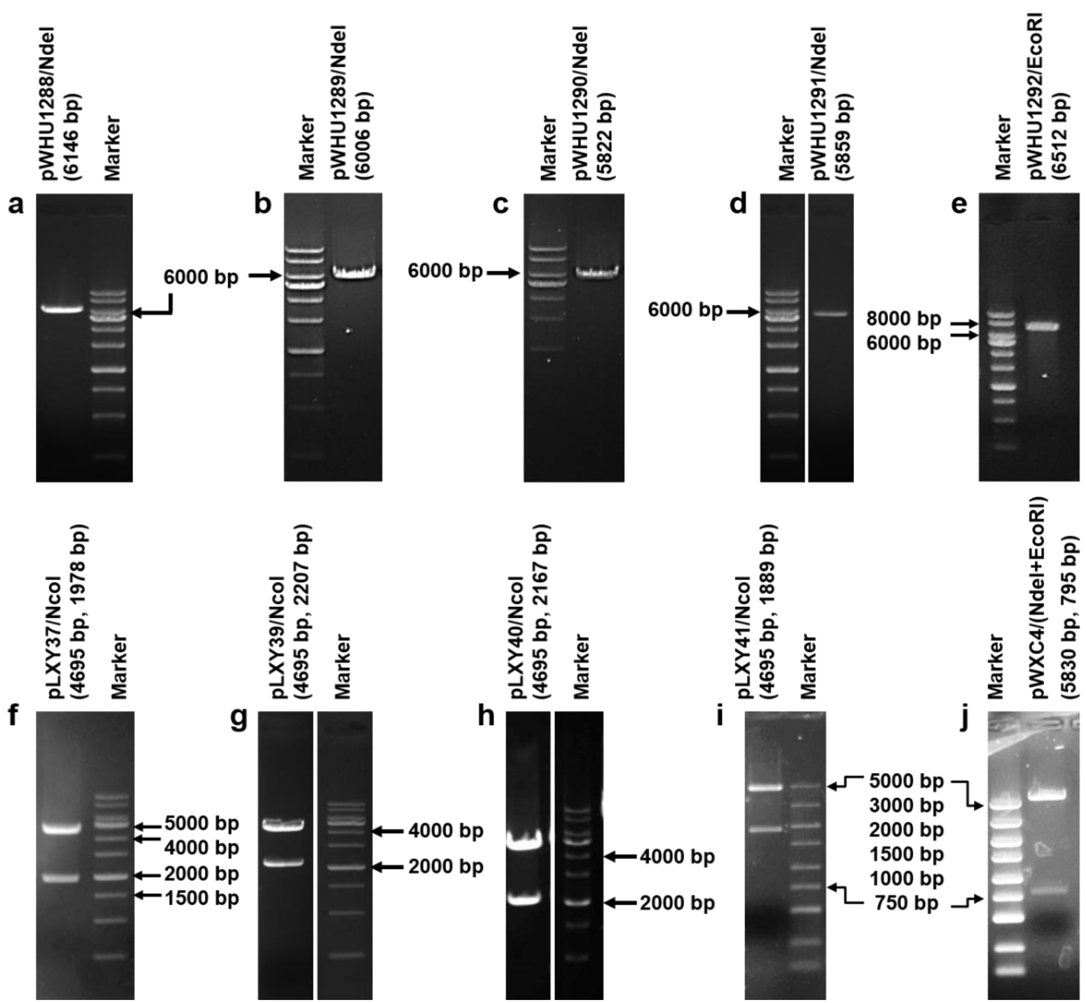

Figure S2 Agarose gel electrophoresis analysis of recombinant plasmids digested with restriction enzymes. Gels were stained with ethidium bromide and photographed under UV light at 305 nm. All recombinant plasmids were sequenced by TsingKe Inc., Wuhan, China. a, pWHU1288; b, pWHU1289; c, pWHU1290; d, pWHU1291; e, pWHU1292; f, pLXY37; g, pLXY39; h, pLXY40; i, pLXY41; j, pWXC4.

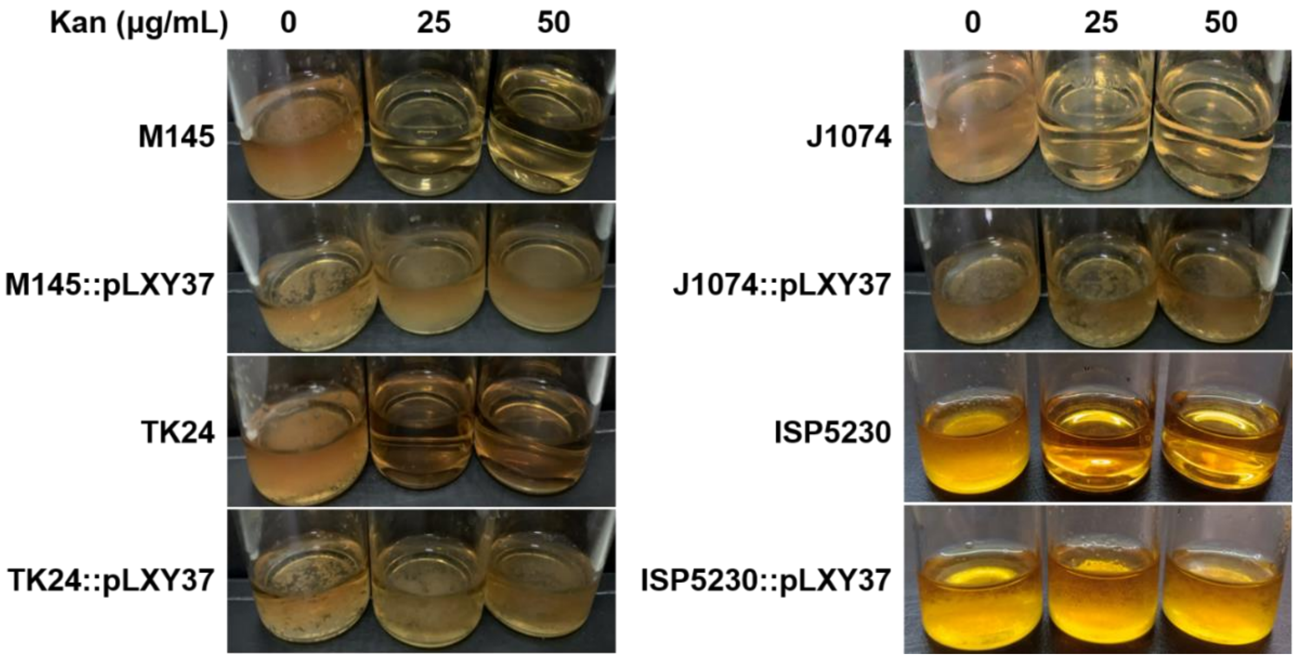

Figure S3 Determination of the activity of kanamycin resistance gene *neo* controlled by the promoter *ermE*p* in different *Streptomyces* strains. The wild type strains including *S. coelicolor* M145, *S. lividans* TK24, *S. albus* J1074 and *S. venezuelae* ISP5230 were controls. The corresponding recombinant strains harboring pLXY37 inoculated in the liquid TSBY medium with different concentrations of kanamycin and cultured at 30 °C and 200 rpm for 48 h.

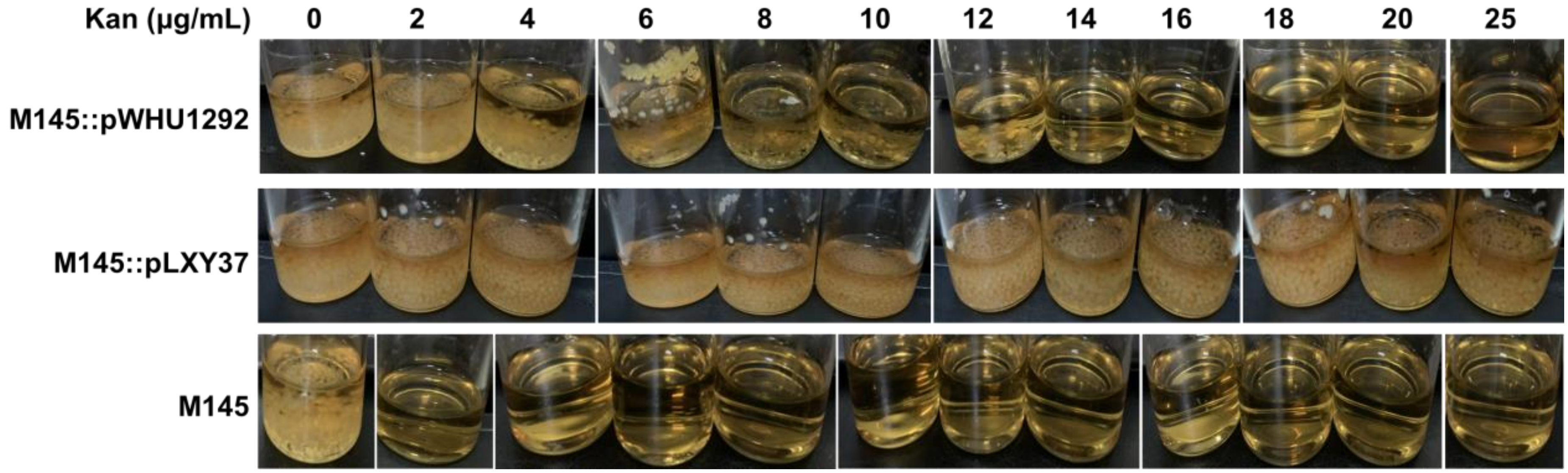

Figure S4 Determination of the activity of promoterless kanamycin resistance gene *neo* in *S. coelicolor* M145::pWHU1292. *S. coelicolor* M145 and M145::pLXY37 were controls. These strains were inoculated in TSBY liquid medium containing different concentrations of kanamycin and cultured at 30°Cand 200 rpm for 48 h.

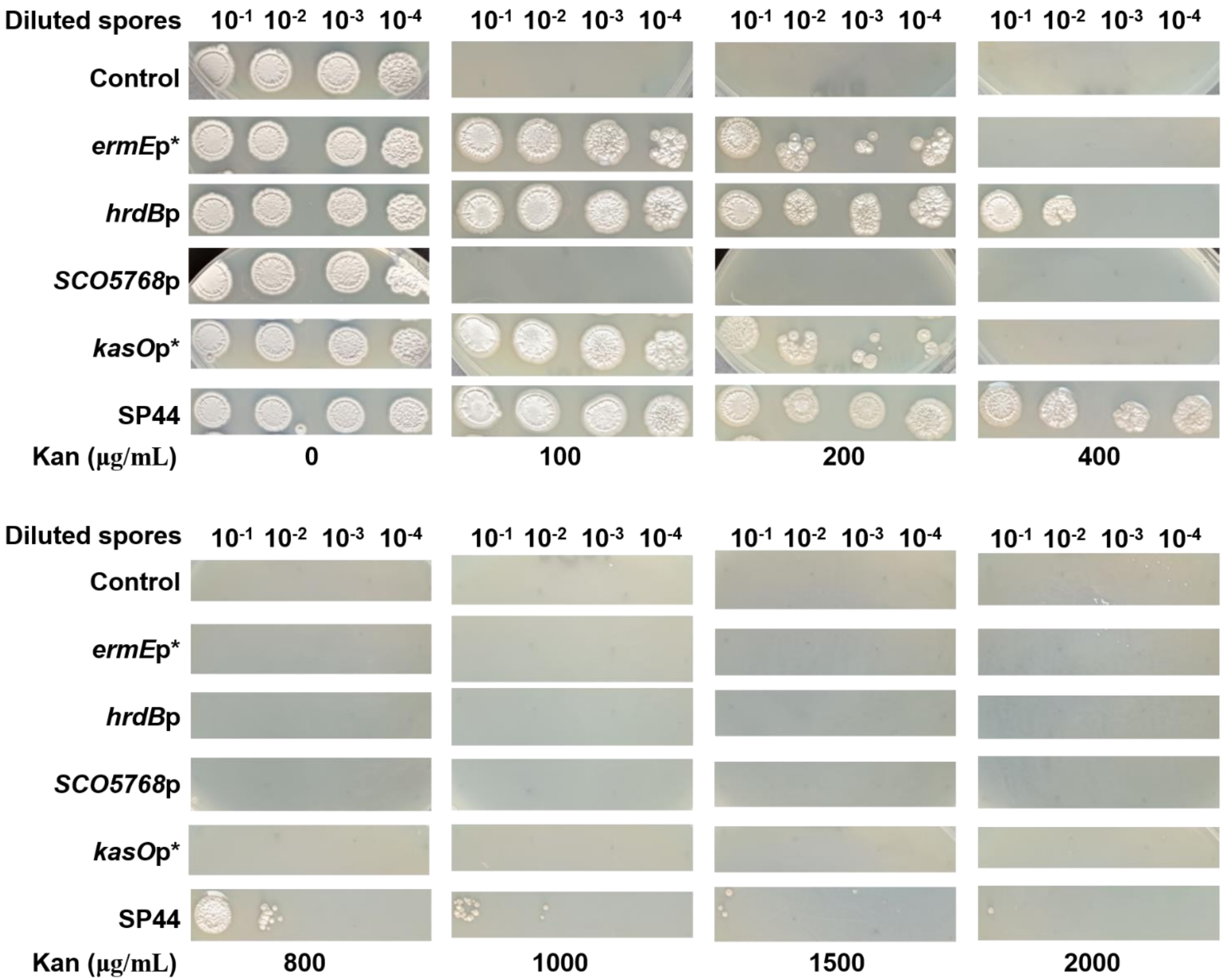

Figures S5: Determination of promoter activity by using *neo* as a reporter gene in *S. coelicolor* M145. （SFM, 30℃, Day8）

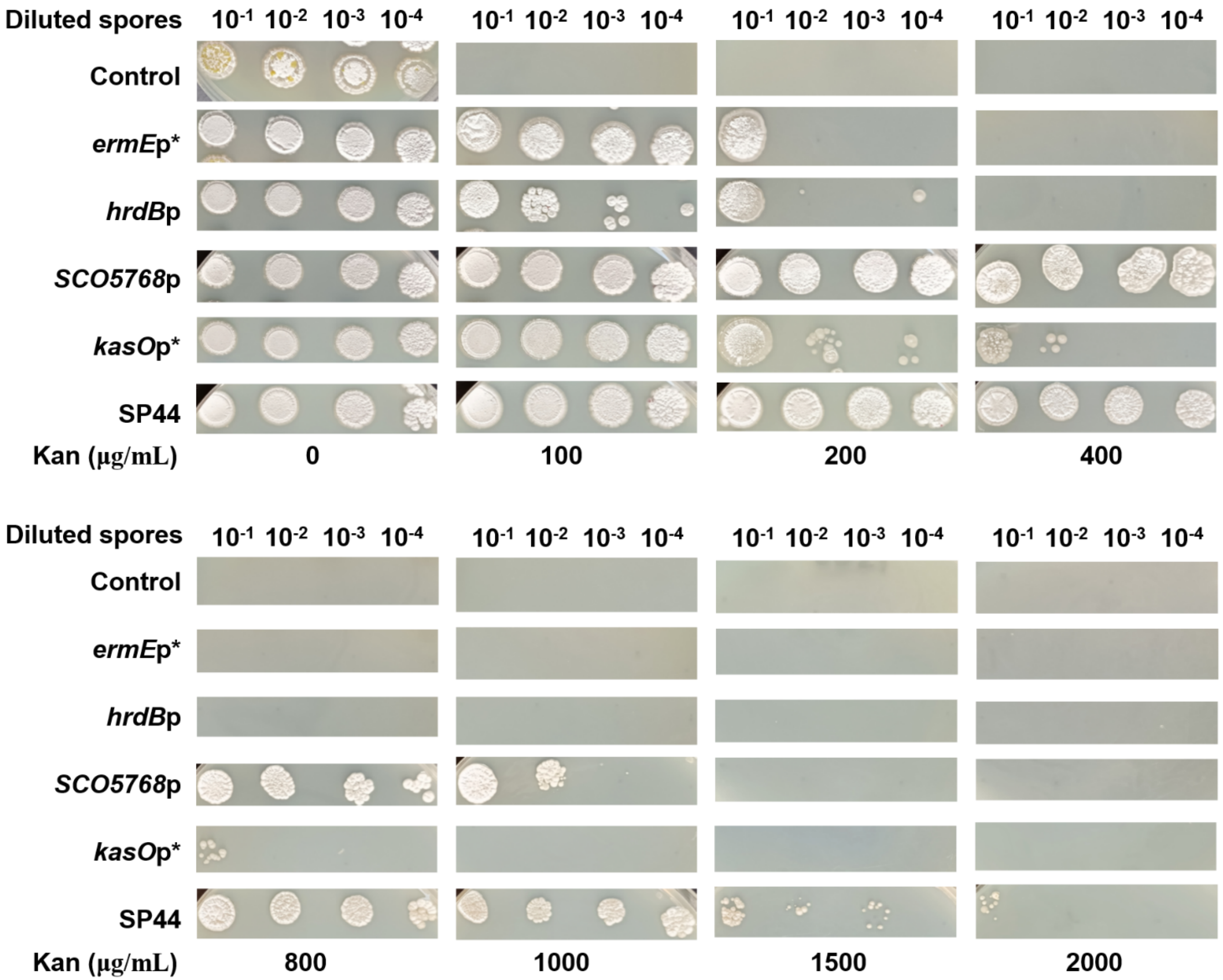

Figures S6: Determination of promoter activity by using *neo* as a reporter gene in *S. lividans* TK24 （SFM, 30℃, Day8）

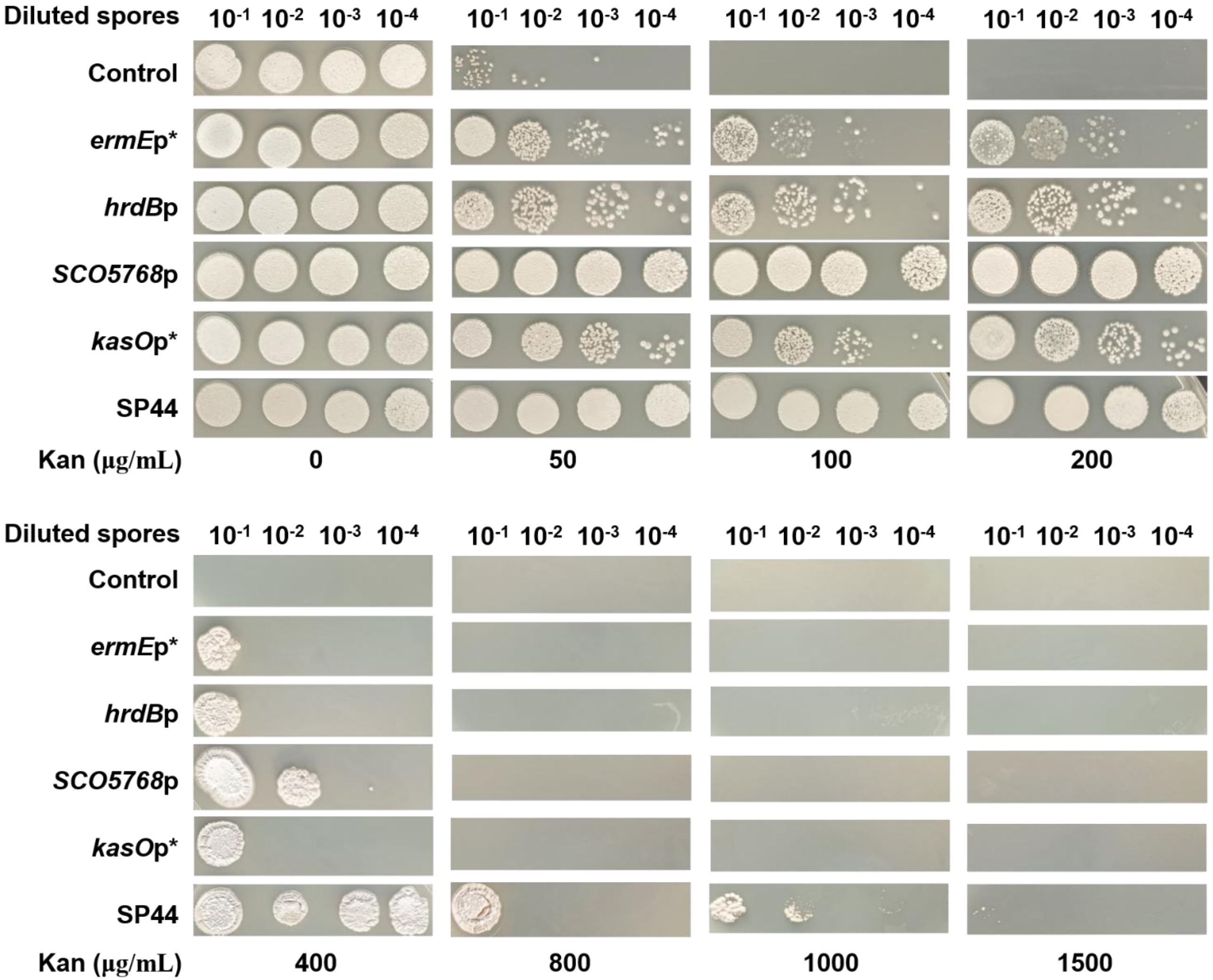

Figures S7: Determination of promoter activity by using *neo* as a reporter gene in *S. olivaceus* CGMCC 4.1369 （SFM, 30℃, Day8）

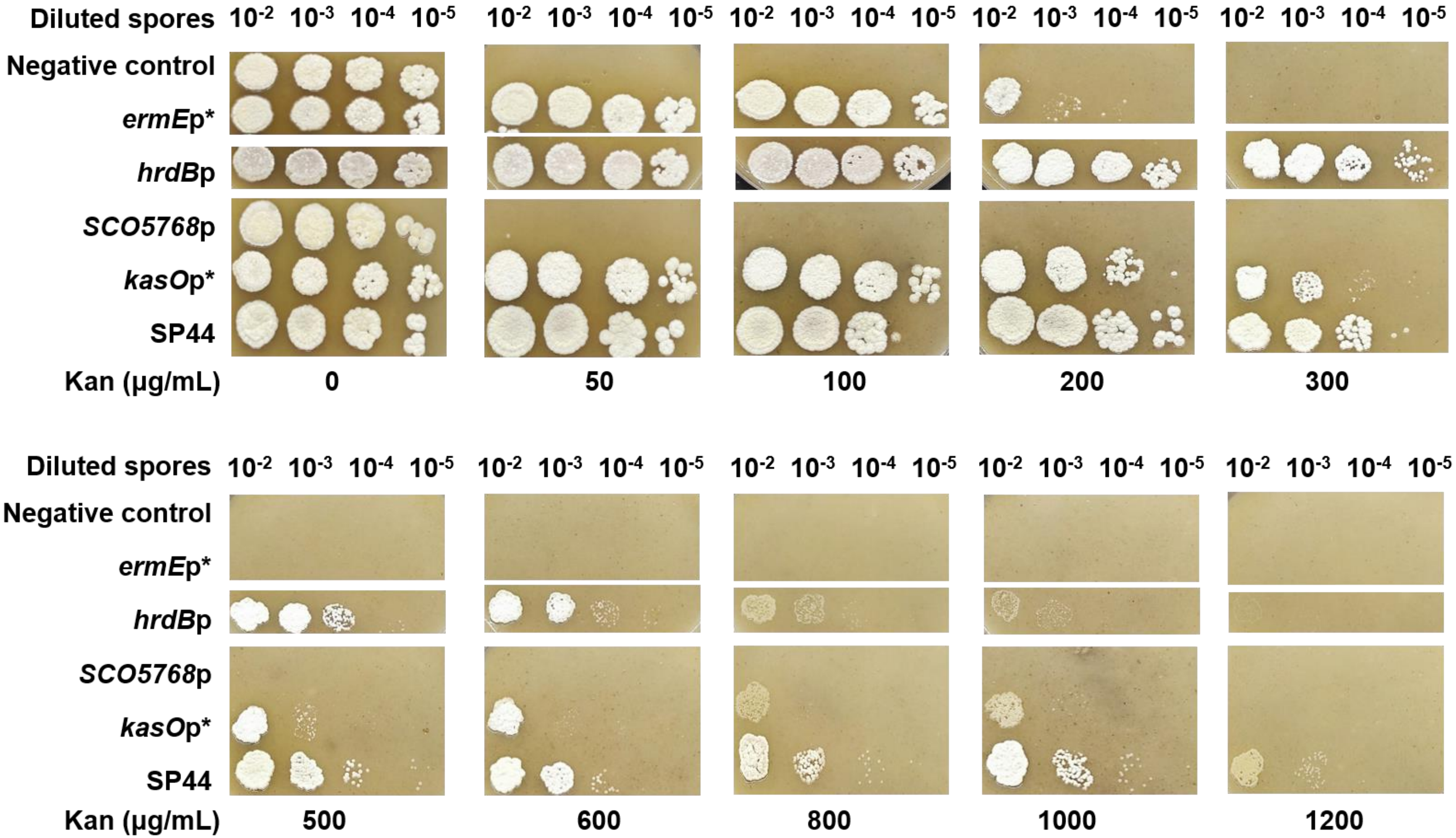

Figures S8: Determination of promoter activity by using *neo* as a reporter gene in *Streptomyces* sp. 219807 （FM, 30℃, Day 7）

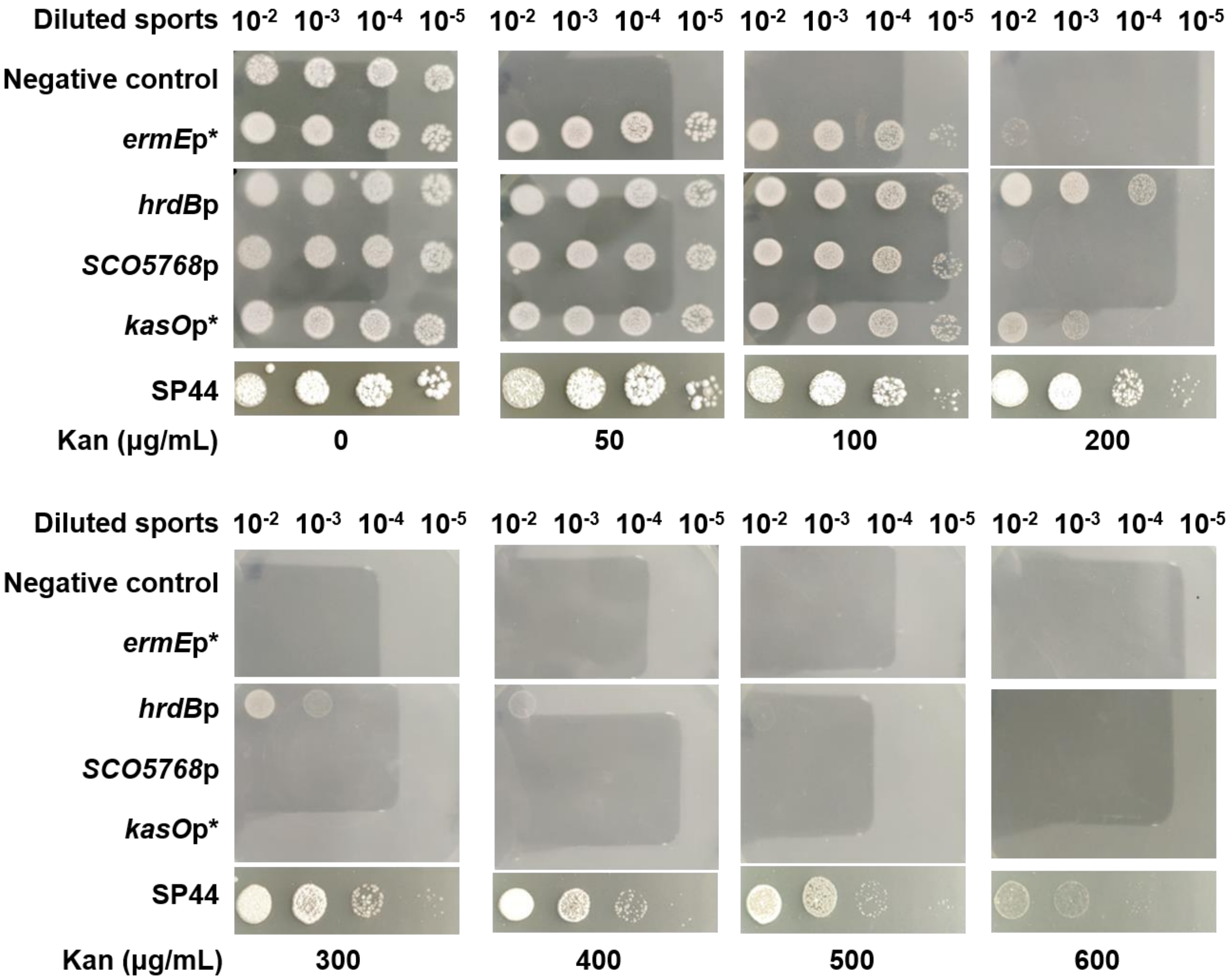

Figures S9: Determination of promoter activity by using *neo* as a reporter gene in *Streptomyces* sp. 211726 （FM, 30℃, Day 6）

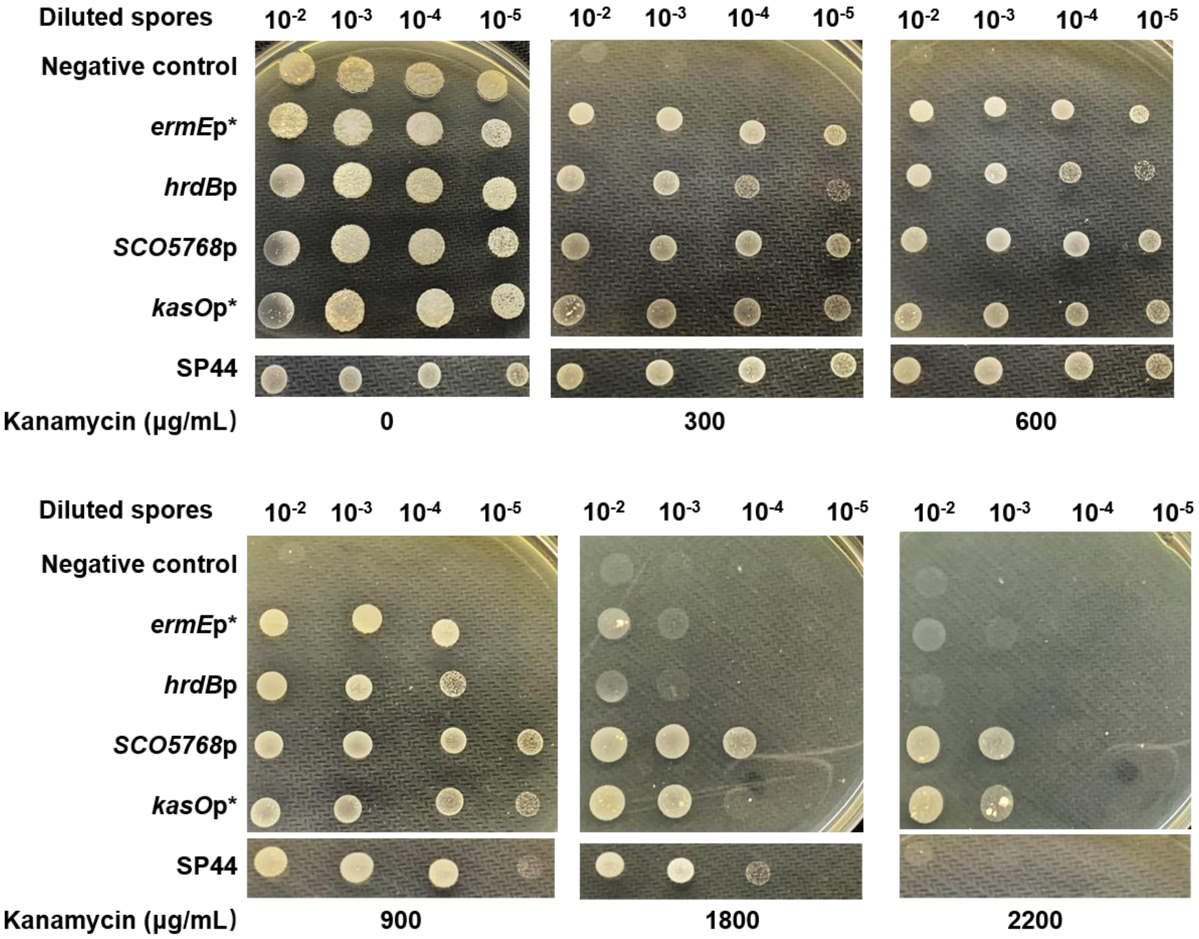

Figures S10: Determination of promoter activity by using *neo* as a reporter gene in *S. armeniacus* DSM 43125（FM, 30°C, Day 8）

Table S3: Deduced functions and sequence comparison of elaiophylin biosynthetic genes

Name	Size (aa)	Proposed function	Homolog (aa, identity% /similarity%) [strain]
Ela6	481	lipase maturation factor family protein	WP_086883949.1 (481, 98/98) [*S. rhizosphaericus*]
Ela5	346	beta-ketoacyl-ACP synthase III	WP_044567923.1 (346, 96/97) [*S. iranensis*]
Ela4	571	3-hydroxyacyl-CoA dehydrogenase	WP_086883947.1 (572, 98/98) [*S. rhizosphaericus*]
Ela3	958	AAA family ATPase and lux regulon	WP_086883946.1 (958, 99/99) [*S. rhizosphaericus*]
Ela2	325	glucose-1-phosphate thymidylyltransferase	RLV74943.1 (316, 98/98) [*S. rapamycinicus* NRRL 5491]
Ela1	324	dTDP-glucose 4,6-dehydratase	WP_044567919.1 (324, 98/99) [*S. iranensis*]
ElaA	4561	PKS (KS-AT-ACP-KS-AT-DH-KR-ACP-KS-AT-KR-ACP)	AEM83457.1 (4516, 87/89) [*S. violaceusniger* Tu 4113]
ElaB	1660	PKS (KS-AT-KR-ACP)	WP_037957959.1 (1708, 91/93) [*Streptomyces* sp. PRh5]
ElaC	1648	PKS (KS-AT-KR-ACP)	WP_138910801.1 (1677, 95/96) [*Streptomyces* sp. DASNCL29]
ElaD	3388	PKS (KS-AT-KR-ACP-KS-AT-DH-KR-ACP)	WP_037962578.1 (3386, 97/97) [*Streptomyces* sp. PRh5]
ElaE	2100	PKS (KS-AT-DH-KR-ACP-TE)	WP_138910799.1 (2088, 96/97) [*Streptomyces* sp. DASNCL29]
Ela1*	263	Type II TE	WP_044567914.1 (261, 97/99) [*S. iranensis*]
Ela2*	417	activator-dependent family glycosyltransferase	WP_138910797.1 (417, 98/98) [*Streptomyces* sp. DASNCL29]
Ela3*	198	dTDP-4-keto-6-deoxy-D-glucose epimerase	WP_086883282.1 (198, 98/98) [*Streptomyces* sp. DASNCL29]
Ela4*	304	ABC transporter ATP-binding protein	WP_020866317.1 (304, 100/100) [*Streptomyces*]
Ela5*	245	ABC transporter permease	WP_037962594.1 (245, 100/100) [*Streptomyces*]
Ela6*	420	sensor histidine kinase	EXU62917.1 (420, 97/97) [*Streptomyces* sp. PRh5]
Ela7*	222	DNA-binding response regulator	WP_138910795.1 (222, 99/100) [*Streptomyces* sp. DASNCL29]
Ela8*	321	NAD(P)-dependent oxidoreductase	WP_037962600.1 (321, 97/97) [*Streptomyces* sp. PRh5]
Ela9*	328	aldo/keto reductase	WP_138910793.1 (328, 99/99) [*Streptomyces* sp. DASNCL29]
Ela10*	468	NDP-hexose 2,3-dehydratase	WP_086879683.1 (469, 99/99) [*S. rhizosphaericus*]
Ela11*	446	crotonyl-CoA carboxylase/reductase	WP_086879681.1 (446, 99/99) [*S. rhizosphaericus*]
Ela12*	449	peroxidase	WP_138910790.1 (460, 90/91) [*Streptomyces* sp. DASNCL29]
Ela13*	288	Fpg/Nei family DNA glycosylase	WP_138910789.1 (288, 99/99) [*Streptomyces* sp. DASNCL29]

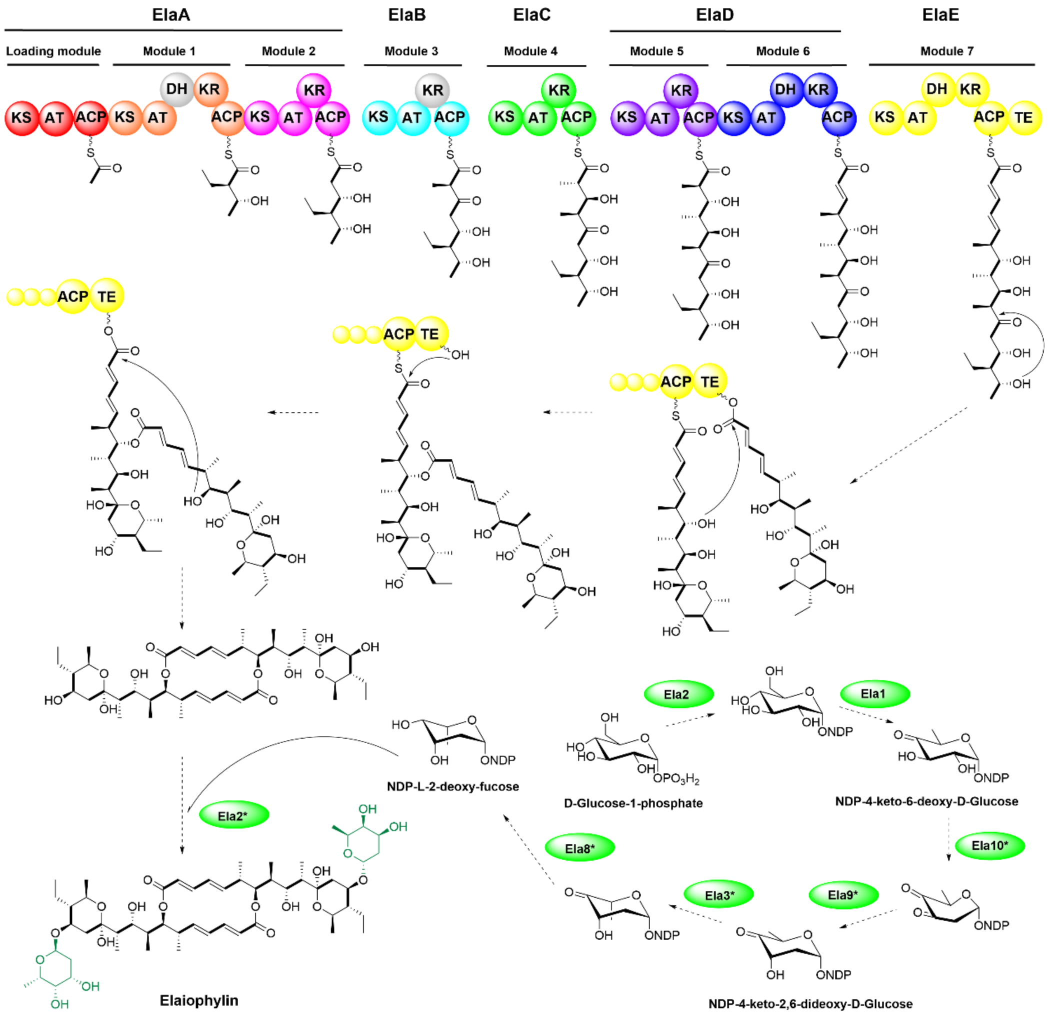

Figure S11 Proposed biosynthetic pathway of elaiophylin

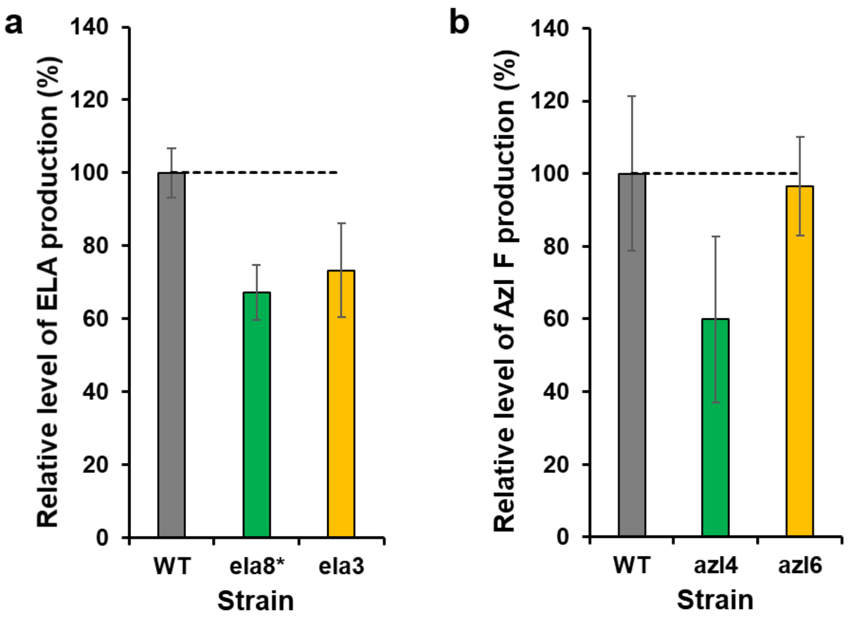

Figure S12 Relative levels of elaiophylin productions by *Streptomyces* sp. 219807 derivative strains detected and quantified by HPLC (a): ELA, elaiophylin; WT, 219807::pWHU1291; *ela8**, 219807::pMX401; *ela3*, 219807::pMX405. Relative levels of azalomycin F productions by *Streptomyces* sp. 211726 derivative strains detected and quantified by HPLC (b): Azl F, azalomycin F mixtures; WT, 211726::pWHU1291; *azl4*, 211726::pMX404; *azl6*, 211726::pMX403. Error bars indicate the standard deviation (n=3)

Reference

1. Qiao, Y.; Yan, J.; Jia, J.; Xue, J.; Qu, X.; Hu, Y.; Deng, Z.; Bi, H.; Zhu, D., Characterization of the Biosynthetic Gene Cluster for the Antibiotic Armeniaspirols in *Streptomyces armeniacus*. *Journal of natural products* **2019,** 82, (2), 318-323.
2. Han, Y.; Tian, E.; Xu, D.; Ma, M.; Deng, Z.; Hong, K., Halichoblelide D, a New Elaiophylin Derivative with Potent Cytotoxic Activity from Mangrove-Derived *Streptomyces* sp. 219807. *Molecules* **2016,** 21, (8).
3. Yuan, G.; Lin, H.; Wang, C.; Hong, K.; Liu, Y.; Li, J., 1H and 13C assignments of two new macrocyclic lactones isolated from *Streptomyces* sp. 211726 and revised assignments of azalomycins F3a, F4a and F5a. *Magnetic resonance in chemistry : MRC* **2011,** 49, (1), 30-7.
4. Xu, W.; Zhai, G.; Liu, Y.; Li, Y.; Shi, Y.; Hong, K.; Hong, H.; Leadlay, P. F.; Deng, Z.; Sun, Y., An Iterative Module in the Azalomycin F Polyketide Synthase Contains a Switchable Enoylreductase Domain. *Angewandte Chemie* **2017,** 56, (20), 5503-5506.
5. Paget, M. S.; Chamberlin, L.; Atrih, A.; Foster, S. J.; Buttner, M. J., Evidence that the extracytoplasmic function sigma factor sigmaE is required for normal cell wall structure in *Streptomyces coelicolor* A3(2). *J Bacteriol* **1999,** 181, (1), 204-11.
6. MacNeil, D. J.; Gewain, K. M.; Ruby, C. L.; Dezeny, G.; Gibbons, P. H.; MacNeil, T., Analysis of *Streptomyces avermitilis* genes required for avermectin biosynthesis utilizing a novel integration vector. *Gene* **1992,** 111, (1), 61-8.
7. Sun, Y.; He, X.; Liang, J.; Zhou, X.; Deng, Z., Analysis of functions in plasmid pHZ1358 influencing its genetic and structural stability in *Streptomyces lividans* 1326. *Applied microbiology and biotechnology* **2009,** 82, (2), 303-10.
8. Bierman, M.; Logan, R.; O'Brien, K.; Seno, E. T.; Rao, R. N.; Schoner, B. E., Plasmid cloning vectors for the conjugal transfer of DNA from *Escherichia coli* to Streptomyces spp. *Gene* **1992,** 116, (1), 43-9.
9. Wilkinson, C. J.; Hughes-Thomas, Z. A.; Martin, C. J.; Bohm, I.; Mironenko, T.; Deacon, M.; Wheatcroft, M.; Wirtz, G.; Staunton, J.; Leadlay, P. F., Increasing the efficiency of heterologous promoters in actinomycetes. *Journal of molecular microbiology and biotechnology* **2002,** 4, (4), 417-26.
10. Wang, W.; Li, X.; Wang, J.; Xiang, S.; Feng, X.; Yang, K., An engineered strong promoter for streptomycetes. *Applied and environmental microbiology* **2013,** 79, (14), 4484-92.
11. Zhang, B.; Tian, W.; Wang, S.; Yan, X.; Jia, X.; Pierens, G. K.; Chen, W.; Ma, H.; Deng, Z.; Qu, X., Activation of Natural Products Biosynthetic Pathways via a Protein Modification Level Regulation. *ACS Chem Biol* **2017,** 12, (7), 1732-1736.
12. Wang, T.; Bai, L.; Zhu, D.; Lei, X.; Liu, G.; Deng, Z.; You, D., Enhancing macrolide production in Streptomyces by coexpressing three heterologous genes. *Enzyme Microb Technol* **2012,** 50, (1), 5-9.
13. Du, D.; Zhu, Y.; Wei, J.; Tian, Y.; Niu, G.; Tan, H., Improvement of gougerotin and nikkomycin production by engineering their biosynthetic gene clusters. *Applied microbiology and biotechnology* **2013,** 97, (14), 6383-96.
